# repDilPCR: a tool for automated analysis of qPCR assays by the dilution-replicate method

**DOI:** 10.1186/s12859-024-05954-9

**Published:** 2024-10-15

**Authors:** Deyan Yordanov Yosifov, Michaela Reichenzeller, Stephan Stilgenbauer, Daniel Mertens

**Affiliations:** 1 Division of CLL, Department of Internal Medicine III, Ulm University Hospital, Ulm, Germany; 2 Cooperation Unit “Mechanisms of Leukemogenesis”, German Cancer Research Center (DKFZ), Heidelberg, Germany; 3 Comprehensive Cancer Center Ulm (CCCU), Ulm University Hospital, Ulm, Germany

**Keywords:** QPCR, Dilution-replicate design, RT-PCR, Polymerase chain reaction, Automation, Plotting, Statistics, Web server, R, Shiny

## Abstract

**Background:**

The dilution-replicate experimental design for qPCR assays is especially efficient. It is based on multiple linear regression of multiple 3-point standard curves that are derived from the experimental samples themselves and thus obviates the need for a separate standard curve produced by serial dilution of a standard. The method minimizes the total number of reactions and guarantees that Cq values are within the linear dynamic range of the dilution-replicate standard curves. However, the lack of specialized software has so far precluded the widespread use of the dilution-replicate approach.

**Results:**

Here we present repDilPCR, the first tool that utilizes the dilution-replicate method and extends it by adding the possibility to use multiple reference genes. repDilPCR offers extensive statistical and graphical functions that can also be used with preprocessed data (relative expression values) obtained by usual assay designs and evaluation methods. repDilPCR has been designed with the philosophy to automate and speed up data analysis (typically less than a minute from Cq values to publication-ready plots), and features automatic selection and performance of appropriate statistical tests, at least in the case of one-factor experimental designs. Nevertheless, the program also allows users to export intermediate data and perform more sophisticated analyses with external statistical software, e.g. if two-way ANOVA is necessary.

**Conclusions:**

repDilPCR is a user-friendly tool that can contribute to more efficient planning of qPCR experiments and their robust analysis. A public web server is freely accessible at https://repdilpcr.eu without registration. The program can also be used as an R script or as a locally installed Shiny app, which can be downloaded from https://github.com/deyanyosifov/repDilPCR where also the source code is available.

**Supplementary Information:**

The online version contains supplementary material available at 10.1186/s12859-024-05954-9.

## Background

**Fig. 1:**
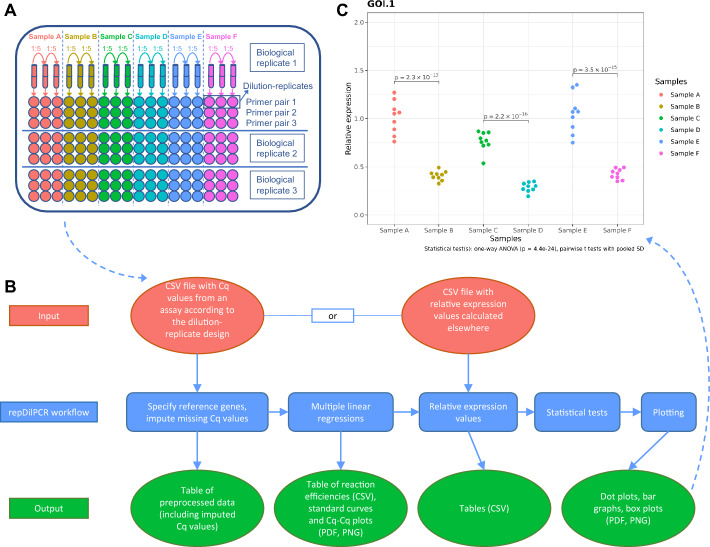
**A** Reaction setup according to the dilution-replicate design. **B** repDilPCR workflow. **C** An example of a completely automatically created plot from experimental data. The final plot displays 9 biological replicates per sample although only 3 biological replicates could be shown on the diagram in (**A**) for space reasons

Determination of polymerase chain reaction (PCR) efficiency for each primer pair is of key importance for correct evaluation and interpretation of quantitative PCR (qPCR) data [1–3]. Different approaches to determine efficiency have been developed, from the classical standard curve-based method to sophisticated methods that rely on fitting linear or non-linear models on individual amplification curves [4–7]. Occupying the middle ground between these two extremes is the dilution-replicate experimental design [8] that has remained underused, most probably due to the lack up to now of a dedicated software to apply the method. The dilution-replicate approach is based on multiple linear regression and offers a number of advantages. It requires fewer reactions and thus helps to reduce costs. In the traditional approach, standard curves are produced by a separate set of dilutions of a standard sample. In the dilution-replicate design (Fig. 1A), standard curves are determined from so-called dilution-replicates of experimental samples that serve both to control technical variance and to determine efficiency [8]. In this way, all samples contribute to the efficiency estimate and precision increases with the number of samples on a plate. Furthermore, the traditional approach requires that the linear dynamic range of the independent standard curve covers all sample Cq values. This requirement sometimes makes it necessary to repeat experiments using different dilutions. In contrast, with the dilution-replicate design it is guaranteed that the sample Cq values will be within range.

The theoretical basis of the dilution-replicate method, including derivation of mathematical formulas, estimation of reaction efficiency by collinear fit of standard curves through multiple linear regression and estimation of relative changes in gene expression from Cq-Cq plots are described in the original article [8] and potential users of the method are advised to read it. For convenience, we have included the most important formulas in the Supplementary Information (Additional file 1). From a practical standpoint, the experimenter applying the dilution-replicate method should prepare three tubes with serial dilutions (usually fivefold) of each cDNA preparation. Three wells on a qPCR plate are necessary for every biological replicate / primer pair combination, analogous to the number of wells that one needs for technical replicates with the traditional approach. However, with the dilution-replicate method the amount of cDNA in each well is not the same but step-wise reduced (same volume from the different dilutions prepared in the tubes). No wells are necessary for separate standard curves. Control reactions in which no amplification is expected (no template, no reverse transcription and genomic DNA controls) should be performed with identical replicates as usual. It should be noted that analogous to the classical standard curve method, the dilution steps for the dilution-replicate method should be performed with a properly calibrated pipette, otherwise a bias would be introduced into the calculated efficiencies and relative quantities.

Our tool, repDilPCR, utilizes the described dilution-replicate analytical method [8] and extends it by adding the possibility to use multiple reference genes, a prerequisite for accurate qPCR expression profiling [9]. It also offers capabilities for performing statistical tests and plotting publication-ready graphs. The program has been designed with the philosophy to automate and speed up analysis of qPCR data (typically less than one minute from Cq values to publication-ready plots) and to help users select and perform the appropriate statistical tests, at least in the case of one-factor experimental designs. At the same time, the program allows experienced users to export intermediate data and perform more sophisticated analyses with external statistical software, e.g. if two-way analysis of variance (ANOVA) is necessary.

Although the primary goal of the program is to enable analysis of qPCR data via the dilution-replicate approach, the statistical and plotting functions can also be used with preprocessed data, i.e. with relative expression values obtained by usual assay designs and evaluation methods.

## Implementation

repDilPCR is written in R/Shiny and can be used both as an ordinary R script on a local computer or as a Shiny app (either on a local computer or on a server) accessed through a web browser. A publicly available instance of the Shiny app is hosted at the German Cancer Research Center (DKFZ) in Heidelberg (https://repdilpcr.eu). This service is anonymous, does not require registration and complies with common standards for protection of user data: raw data uploaded by the user are processed on the server and used to generate results that can be downloaded by the user; after the user closes the session by closing the browser window all uploaded data and processed results are automatically deleted from the server (Warning: if you use a local installation of repDilPCR, do not store your data in the folder where repDilPCR is installed or they will be deleted!).

The source code of the program is organized in three separate files. repDilPCR_lib.R is the core of the program. It is a library of functions that is used by the other two scripts: app.R (the Shiny app) and repDilPCR.R (the executable R script) that can function independently of each other. Further details and installation instructions for users that would like to install the program locally are available in the Supplementary Information (Additional file 1), as well as on the GitHub page of the project: https://github.com/deyanyosifov/repDilPCR.

## Features and usage

The workflow is summarized in Fig. 1B. repDilPCR has been designed with the philosophy to automate and speed up data analysis. Once the input data have been uploaded in the correct format, the user can achieve all of the following with just a few clicks and within 1–2 min:

- Impute missing Cq values for reference genes (using the weighted predictive mean matching method from the R package mice [10]),
- Perform multiple linear regressions to get standard curves and Cq-Cq plots for all amplicons (based on Eq. 3 and 5 from the original article describing the dilution-replicate approach [8]),
- Identify possible outliers,
- Calculate relative quantities of the templates,
- Perform statistical tests to compare experimental groups,
- Prepare publication-ready plots (as in Fig. 1C),
- And download the results in a suitable format: Comma-Separated Values (CSV), Portable Document Format (PDF) or Portable Network Graphics (PNG).

### Preparation of input data

This preparatory step is the same no matter whether one intends to use the R script or the Shiny app.

Input data have to be arranged in a CSV file following a specific format depending on the experimental setup and type of data: (a) unprocessed Cq values obtained from an experiment performed according to the dilution-replicate approach, or (b) already calculated relative expression values. Exemplary input data tables for these two use cases are provided in the files Test_data.csv and Test_data_precalc.csv, respectively, which are available in the installation directory or can be downloaded using the buttons on the “About/Help” tab of the repDilPCR program. In the exemplary files, points are used as decimal separators and commas as field separators (to separate values in each row). It is also possible to use commas as decimal separators and semicolons as field separators—the default regional setting in most European countries. The program will recognize the format automatically.

*Input data consisting of unprocessed Cq values (dilution-replicate approach).* It is crucial that a common threshold has to be set for all genes that are being compared in an experiment before exporting the Cq values from the software of the qPCR machine. This is necessary because of the assumptions of the mathematical model derived in the original article and implemented in repDilPCR (see Additional file 1 for a brief summary). Depending on the manufacturer of the machine and the respective software, Cq values might be referred to as Ct ("cycle threshold") or Cp ("crossing point") values but these different names stand for the same concept. Here, we adhere to the Minimum Information for Publication of Quantitative Real-Time PCR Experiments (MIQE) guidelines and the respective terminology (Cq = quantification cycle) [1]. The CSV file needs to have the following layout: The first row contains column titles. The first three columns have predetermined names that must not be changed. The first column is called "Replicates" and it should contain the names of the samples with a suffix that identifies the biological replicate. The suffix consists of an underscore ("_") plus additional numbers and/or letters. The second column is called "Pairs" and can contain optional information about grouping of samples in pairs. The third column is called "Dilution" and contains the dilution factors according to the dilution-replicate design. For example, if the experiment was performed with fivefold serial dilutions, one can use as factors the numbers 1, 5 and 25. The following columns should contain the Cq values for the assessed genes, first the reference genes (RG) and then the genes of interest (GOI). The titles of these columns should be the names of the respective genes/amplicons. See the Supplementary Information (Additional file 1) for further details.

*Input data consisting of relative expression values.* In this case, the CSV file that has to be prepared has a simpler layout. Again, the first row contains column titles but now only the first two columns are obligatory and with predetermined names that must not be changed: "Replicates" and "Pairs". Their specification is the same as in the case when Cq values are used (see above). The next columns should contain the relative expression levels (linearly scaled) of the evaluated genes of interest in each biological replicate. Accordingly, the titles of these columns should be the respective gene/amplicon names. See the Supplementary Information (Additional file 1) for further details.

### Usage of the Shiny app

The Shiny app can be used via any modern web browser. Users can:

- Access a publicly available Shiny server with repDilPCR installed on it, for example the installation hosted at the German Cancer Research Center (DKFZ) in Heidelberg (https://repdilpcr.eu), or
- Issue the following commands in the R environment:

﻿﻿﻿﻿library(shiny)

runApp("~/repDilPCR/app.R", launch.browser = TRUE)

replacing the “ ~ /repDilPCR” part with the actual path to their installation, if deviating. This will launch the program and automatically start a new browser window or tab to access it.

The workflow includes the following steps:

1. Upload of properly formatted data
2. Selection of reference genes and (optionally) imputation of missing Cq values (this whole step is only relevant when working with Cq values. Users of the imputation function should read chapter 3.2.2 of Additional file 1 and keep in mind that imputation of too many missing values may lead to erroneous results.)
3. Data analysis
4. Checking the results of the regression analysis (only relevant when working with Cq values, see Additional file 1: Figs. S2 and S3)
5. Visualization of the results. Different types of plots will be available in the graphical interface depending on the chosen settings (Additional file 1: Figs. S4–S8). Possible choices are “Dot plots (all points)”, “Dot plots (means and standard deviations)”, “Bar graphs (means and standard deviations)” and “Box plots”. Graphical parameters like font size, colour scheme, significance symbols, spacing of significance bars, size and resolution of images can be adjusted from the control panel.
6. Statistical tests. repDilPCR aims to make the process of testing statistical hypotheses easy even for users without much knowledge of statistics by automatically selecting appropriate statistical tests depending on the context and properties of the data. The user can choose the broad type of statistical test (parametric or non-parametric) and the comparisons to be tested for statistically significant differences (“all to one (all to reference)”, “all pairs” and “selected pairs”) by clicking on the respective radio buttons in the control panel. The significance level (α) can be freely selected. To make usage of parametric tests possible, all statistical tests are performed on logarithmically transformed data, even when the user chooses to display plots in linear scale (qPCR data are not normally distributed on a linear scale [11]). Comparisons for which the expression of a given gene of interest is significantly different between the groups will be automatically denoted by p-values or asterisks depending on the user's choice. The statistical tests that were performed in each particular case will be listed below the respective plot.
7. Downloading results. All plots and tables that repDilPCR produces can be downloaded from the “Download results” tab. It has three subtabs: “Plots”, “Tables” and “Intermediate data”. Plots can be downloaded in the PDF or PNG file format. PDF files will be multi-page, meaning that the plots for all genes of interest will be put together in a single file on separate pages. Conversely, each PNG file will contain a single plot (gene) but all plots of a particular type will be grouped together and downloaded as a single ZIP archive. In all cases, downloaded files will have automatically created informative file names that will include the name of the dataset (uploaded data file) and the plot type. Additionally, plots in logarithmic scale will have "log" in their file names. Tables will be downloaded as CSV files.

Further details on each of the steps are given in the Supplementary Information (Additional file 1: Chapter 3.2).

### Usage of the R script

Users with experience in R might prefer to use the script due to the more streamlined workflow: one just has to specify the path to the input data, set preferences for the analysis and then execute the script. All results will be automatically saved in the same directory as the raw data without the need to click around in a graphical interface and to download result files one by one. Detailed description is available in the Supplementary Information (Additional file 1: Chapter 3.3).

## Results and validation

We performed two validation qPCR experiments and analysed them using three different methods in parallel: the dilution-replicate approach with repDilPCR, the classical standard curve approach [2, 12] and LinRegPCR [6]. The standard curve approach uses a dilution series of single samples or of a pool of all samples to construct a standard curve for each amplicon and then determines its PCR efficiency from the slope of the curve according to the equation E = 10^[−1/slope]^ [12]. These efficiencies are then used to determine the relative expression of each GOI versus reference genes according to the mathematical model published by Pfaffl or an equivalent model offered by Roche Diagnostics [2]. In contrast, the LinRegPCR program is based on an algorithm that estimates the baseline by reconstructing the log-linear phase downward from the early plateau phase of the PCR reaction [6]. PCR efficiency values are then determined per sample by fitting a regression line to a subset of data points in the log-linear phase. The relative expression of GOIs can then be estimated as described above for the standard curve approach.

**Fig. 2:**
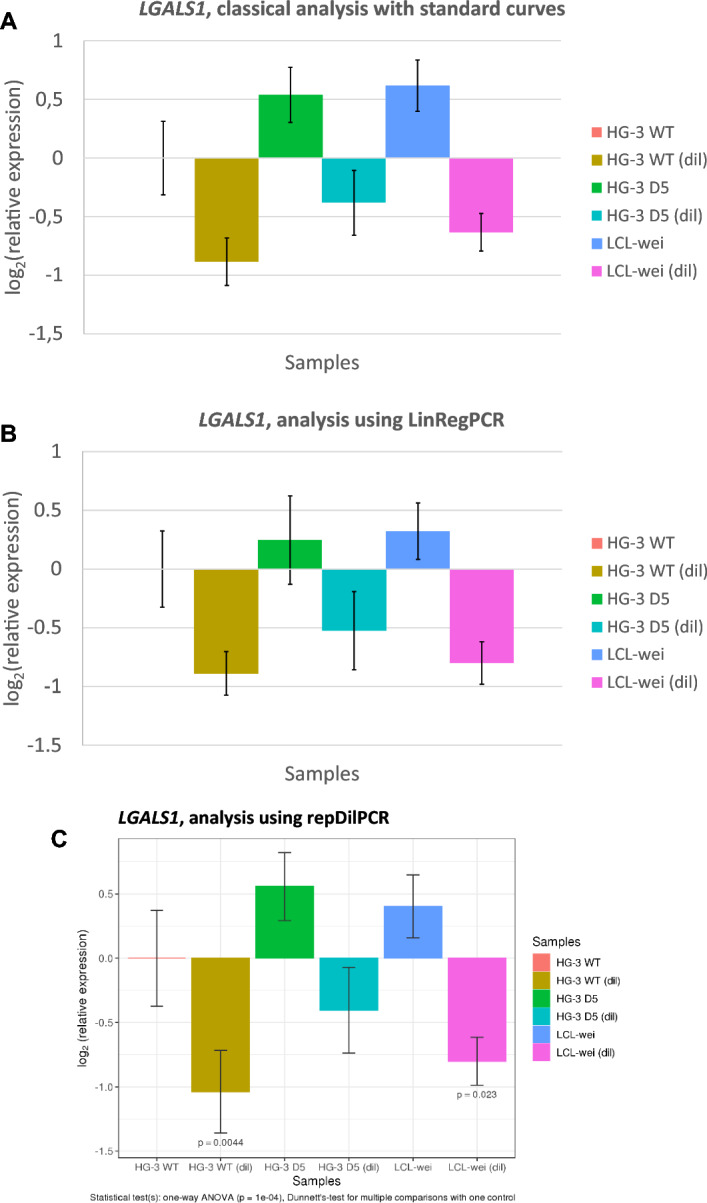
Relative expression of the galectin-1 gene (*LGALS1*) in different cell lines in Validation Experiment 1 as determined according to three different approaches: **A** standard curve; **B** LinRegPCR and **C** repDilPCR. Samples denoted by “(dil)” after the name of the cell line were diluted so that the expression values for *LGALS1* would be half of those in the respective parent samples, i.e. the expected difference is 1 unit on a log_2_ scale (see the Supplementary Data for details). Graphs **A** and **B** were prepared manually using Microsoft Excel. Graph **C** was prepared fully automatically by repDilPCR, starting from unprocessed Cq values. *P*-values are for comparisons with the HG-3 WT sample

**Fig. 3:**
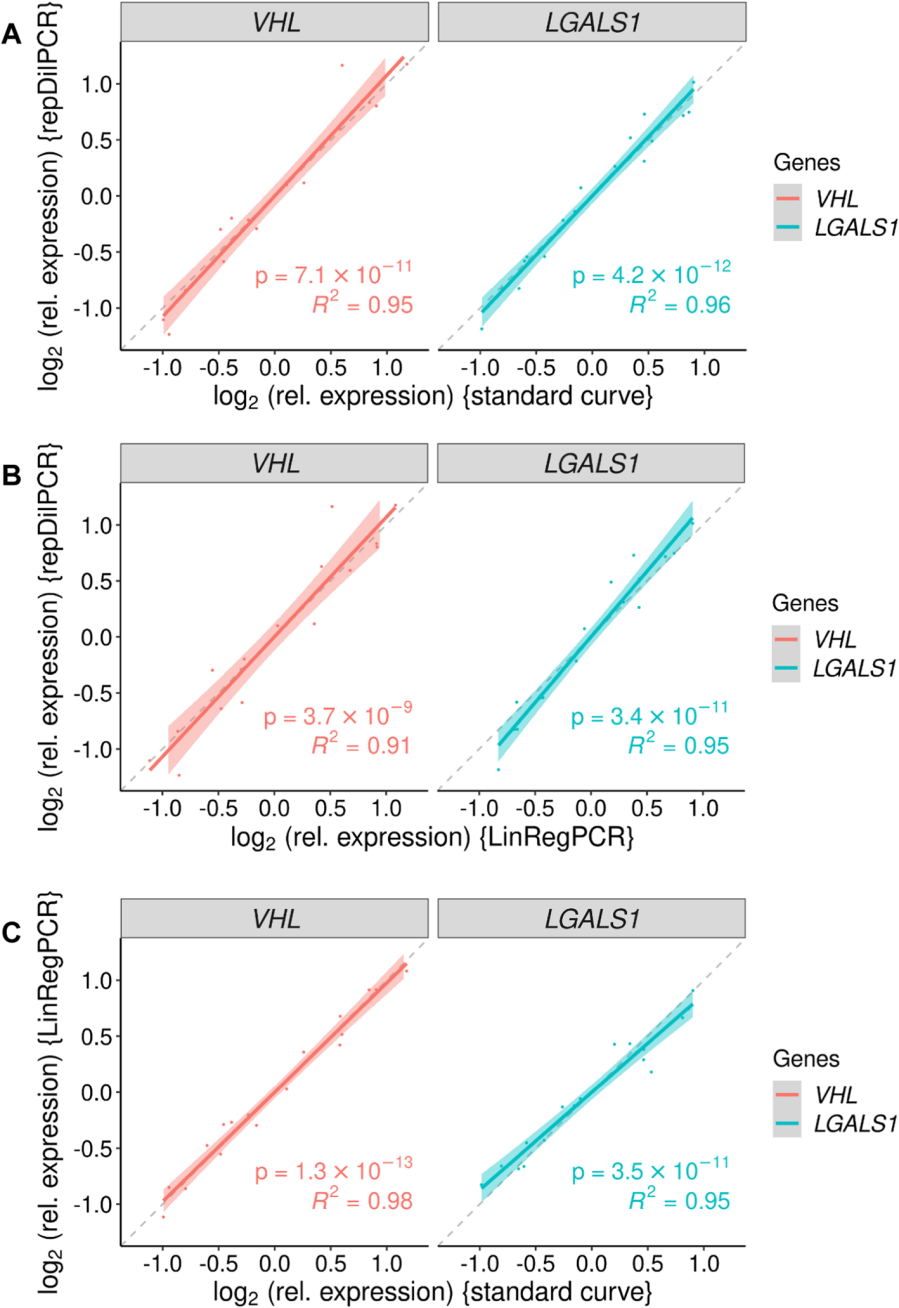
Pairwise scatter plots comparing relative gene expression (on log_2_ scale) determined by three different approaches in Validation Experiment 1. **A** Comparison of repDilPCR against the standard curve method. **B** Comparison of repDilPCR against LinRegPCR. **C** Comparison of LinRegPCR against the standard curve method. Each comparison was performed separately for the two genes of interest that were analysed: *VHL* and *LGALS1*. Each dot corresponds to a biological replicate in the experiment. The data were scaled to set the mean log_2_(relative expression) to 0 to make comparisons possible. Linear regression was performed and the regression line with its 95%-confidence interval is shown for each comparison, as well as the *p*-value and the coefficient of determination (R^2^) of the model. Dashed lines indicate perfect correlation

The comparison of the three analysis methods showed that the obtained results were very similar when mRNA was used as a template in the reverse transcription step (Figs. 2 and 3; Additional file 1: Validation experiment 1). The differences in gene expression between undiluted and 1:1 diluted samples would be expected to be 1 unit on a log_2_ scale and indeed these differences were close to 1 with all of the three analysis approaches and for both investigated GOIs: *LGALS1* (Fig. 2) and *VHL* (Fig. S9). The goodness of the correlation between the methods is demonstrated quantitatively by the high coefficients of determination on the pairwise scatter plots (Fig. 3). Results from repDilPCR correlated a little bit better with results obtained via the standard curve method (R^2^ = 0.95–0.96) than with results from LinRegPCR (R^2^ = 0.91–0.95). Further details are given in the Supplementary Information (Additional file 1: Chapter 5.1, Fig. S9-S11, Table S1).

**Fig. 4:**
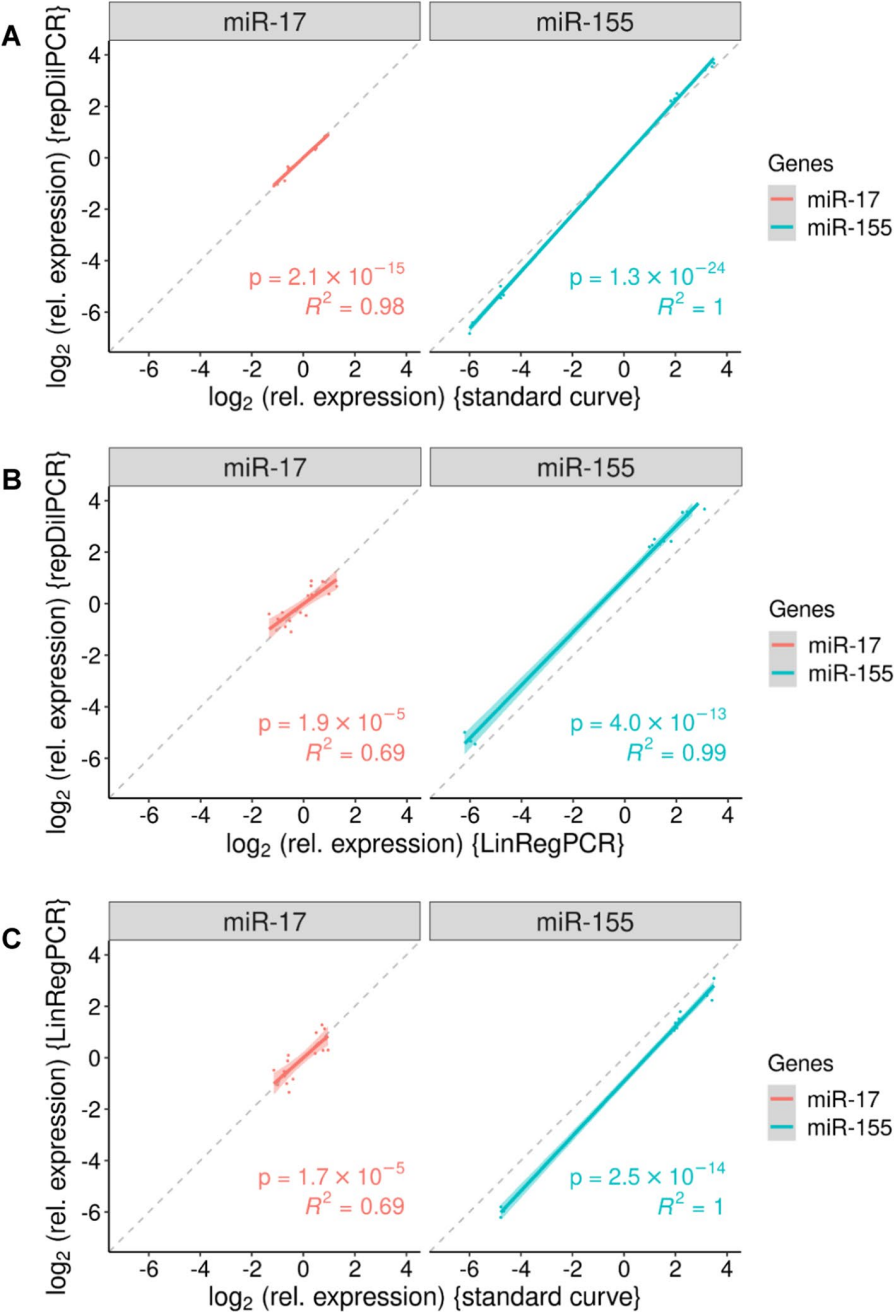
Pairwise scatter plots comparing relative gene expression (on log_2_ scale) determined by three different approaches in Validation Experiment 2. **A** Comparison of repDilPCR against the standard curve method. **B** Comparison of repDilPCR against LinRegPCR. **C** Comparison of LinRegPCR against the standard curve method. Each comparison was performed separately for the two miRNAs that were analysed: miR-17 and miR-155. Each dot corresponds to a biological replicate in the experiment. The data were scaled to set the mean log_2_(relative expression) to 0 to make comparisons possible. Linear regression was performed and the regression line with its 95%-confidence interval is shown for each comparison, as well as the *p*-value and the coefficient of determination (R^2^) of the model. Dashed lines indicate perfect correlation

In the second experiment, the aim was to assess expression levels of miRNAs (Additional file 1: Chapter 5.2, Validation experiment 2). Again, repDilPCR performed similarly to the standard curve method, however LinRegPCR did not always yield satisfactory results, probably because of the flatter amplification curves that made it difficult for the LinRegPCR program to reliably identify windows of linearity (Additional file 1: Fig. S12-S14). Accordingly, results from repDilPCR and the standard curve method correlated very well together (R^2^ = 0.98–1.0, Fig. 4A), whereas correlation was weaker for comparisons of LinRegPCR with the other two methods (Fig. 4B, C): R^2^ was 0.69 for miR-17 and 0.99–1.0 for miR-155 but the regression lines for miR-155 were shifted away from the diagonal because several samples were not evaluable with LinRegPCR and the missing data interfered with the centering of the dataset around zero.

In a third experiment, we verified the reproducibility of the dilution-replicate method by analysing the same samples in three separate PCR runs (Additional file 1: Chapter 5.3, Validation experiment 3, Fig. S15–17, Table S2). Furthermore, we demonstrated successful inter-run normalization using different multi-plate experiment designs—sample maximization or target maximization (Additional file 1: Fig. S18–23).

## Discussion

Here we introduce repDilPCR as a new tool for analysis of qPCR data that brings to the field a number of functions that up to now have not been available in existing software. repDilPCR is the first tool that can make use of the efficient dilution-replicate design for qPCR experiments [8] but is not limited to it and can also work with relative expression values calculated elsewhere. The dilution-replicate approach has important advantages like the guarantee that all samples are within the linear dynamic range of the standard curve and reduced costs due to the possibility to use a smaller number of reactions. We have shown that the results produced by this approach are in good agreement with established gold standard methods (standard curve and LinRegPCR) and hope that its ease of use will stimulate more researchers to abandon bad practices like indiscriminately applying the 2^–∆∆Cq^ method without efficiency corrections or using a single and unvalidated reference gene. The dilution-replicate method has two small disadvantages: it is not suitable for very diluted templates that are close to the limit of detection and it requires slightly more time to prepare the reactions due to the additional dilution steps. However, the last downside is completely offset by the fast analysis of the results when using repDilPCR. Our tool offers a fully automated workflow, including automatic choice of statistical tests to compare experimental groups and generation of publication-ready plots. These functions can also be used with relative expression values calculated by other methods.

Other freely available web servers for analysis of qPCR experiments include qRAT [13], SATQPCR [14], QPCR [15], PIPE-T (as a tool in Galaxy [16]), Auto-qPCR [17] and “Do my qPCR calculation” [18]. Most of these applications use the ∆∆Cq model without efficiency corrections (qRAT, PIPE-T, Auto-q-PCR) or only offer the possibility for manual input of efficiency values calculated elsewhere (SATQPCR, “Do my qPCR calculation”). Only QPCR offers different methods for reaction efficiency determination from the input data. Although all of these tools perform differential expression analysis, the statistical functions in some of them are quite rudimentary and suboptimally implemented, e.g. using parametric tests on non-normally distributed linearly scaled expression values, rather than on log-transformed values (Auto-q-PCR, “Do my qPCR calculation”). These two tools also apply arithmetic averaging of reference genes although geometric averaging should be used. None of the aforementioned tools is able to insert statistical test results directly on the graphical output like repDilPCR. We find a lot of these tools difficult to use as they are not interactive and the user has to download the results before seeing them (SATQPCR, Auto-qPCR, “Do my qPCR calculation”), require uploading separate data files for each sample (PIPE-T) or do not offer an obvious way to group single samples in experimental groups (qRAT). Finally, our attempt to use “Do my qPCR calculation” revealed that the program returns erroneous results because it calculates the delta wrongly as the difference between Cq values of individual wells and the average Cq value for the same gene within an experimental group. On a positive note, some of these tools are able to directly use output files from a number of thermocyclers (qRAT, QPCR, Auto-qPCR) and perform interplate normalization (qRAT, QPCR).

## Conclusions

repDilPCR is an easy-to-use and feature-rich tool for analysis of qPCR experiments that is freely available and can be installed locally or used as a web service. We plan to improve it further, e.g. by adding more helpful hints and error messages. Users can provide feedback and suggest new features in the Discussions tab of the GitHub page of the project.

## Availability and requirements

**Project name:** repDilPCR.

**Project home page:** https://github.com/deyanyosifov/repDilPCR

**Web server:** https://repdilpcr.eu

**Operating system(s):** Platform independent.

**Programming language:** R, Shiny.

**Other requirements:** any modern web browser for accessing the web service; R ≥ 3.6 and R packages (car, gridExtra, tidyverse, mice, PMCMRplus, scales, RColorBrewer, ggbeeswarm, ggsignif, shiny, shinycssloader and shinyalert) for local installations.

**License:** GPL-3, resp. GNU Affero General Public License when installed as server.

**Any restrictions to use by non-academics:** none.

## Supplementary Information

Additional file 1. Supplementary information including further details on method implementation, program installation and usage, as well as validation experiments

## Data Availability

The web server is available at https://repdilpcr.eu, where also example datasets can be downloaded from within the program. The source code can be downloaded from https://github.com/deyanyosifov/repDilPCR, where users can also find answers to frequently asked questions. The datasets used and/or analysed during the current study are available from the corresponding author on reasonable request.

## Abbreviations

**ANOVA**: Analysis of variance
**CSV**: Comma-separated values
**GOI**: Gene(s) of interest
**MIQE**: Minimum information for publication of quantitative real-time PCR experiments
**PCR**: Polymerase chain reaction
**PDF**: Portable Document Format
**PNG**: Portable Network Graphics
**qPCR**: Quantitative polymerase chain reaction
**RG**: Reference gene(s)
