# Supplementary material for: repDilPCR: a tool for automated analysis of qPCR assays by the dilution-replicate method
Source: BMC Bioinformatics. 2024 Oct 15;25:331. doi: 10.1186/s12859-024-05954-9 (PMC11476982; doi:10.1186/s12859-024-05954-9)
Supplement: Supplementary file 1 — Additional file 1. Supplementary information including further details on method implementation, program installation and usage, as well as validation experiments [file 12859_2024_5954_MOESM1_ESM.pdf]

Supplementary Information

repDilPCR: a tool for automated analysis of qPCR assays by the dilution-replicate method

Deyan Y. Yosifov[1,2], Michaela Reichenzeller[1], Stephan Stilgenbauer[1,3] and Daniel Mertens[1,2]

[1] Division of CLL, Department of Internal Medicine III, Ulm University Hospital, Ulm, 89081, Germany; [2] Cooperation Unit "Mechanisms of Leukemogenesis", German Cancer Research Center (DKFZ), Heidelberg, 69120, Germany; [3]Comprehensive Cancer Center Ulm (CCCU), Ulm University Hospital, 89081 Ulm, Germany

1 Implementation ... 2
2 Installation ... 3
2.1 Prerequisites for installation ... 3
2.2 Installation on a local computer ... 3
2.3 Installation on a server ... 3
3 Usage ... 4
3.1 Preparation of input data ... 4
3.2 Usage of the Shiny app ... 6
3.2.1 Upload your data ... 7
3.2.2 Select your reference genes and (optionally) impute missing Cq values ... 7
3.2.3 Analyze your data ... 8
3.2.4 Check the results of the regression analysis ... 8
3.2.5 Visualize the results ... 11
3.2.6 Perform statistical tests ... 11
3.2.7 Adjust the visual appearance of the plots to your needs ... 14
3.2.8 Download results ... 15
3.3 Usage of the R script ... 15
4 Technical notes ... 17
4.1 Imputation of missing Cq values ... 17
4.2 Algorithm for choosing statistical tests ... 17
5 Validation ... 19
5.1 Validation Experiment 1 (comparison with other methods) ... 19
5.1.1 Materials and methods ... 19
5.1.2 Results ... 21
5.2 Validation Experiment 2 (comparison with other methods) ... 25
5.2.1 Materials and methods ... 25
5.2.2 Results ... 26
5.3 Validation Experiment 3 (reproducibility and inter-run calibration) ... 30

5.3.1 Materials and methods 30
5.3.2 Results and discussion 31
6 References 42

1 Implementation

repDilPCR is written in R/Shiny. The source code is organized in three separate files. **`repDilPCR_lib.R`** is the core of the program. It is a library of functions that is used by the other two scripts: **`app.R`** (the Shiny app) and **`repDilPCR.R`** (the executable R script) that can function independently of each other.

repDilPCR analyzes dilution-replicate qPCR data according to the mathematical model provided by the original authors of the method (Hui and Feng, 2013). The model is briefly summarized below.

PCR amplification is described by the equation

$$(1)\ Q(n) = Q(0) \times E^{n}$$

where Q is the quantity of the product, n is the cycle number, E is the efficiency of the reaction and Q(0) represents the initial quantity. For a defined threshold, T, in the exponential phase of the amplification reaction, Cq is defined as the estimated cycle number at which Q crosses T. Consequently,

$$(2)\ T = Q(Cq) = Q(0) \times E^{Cq}$$

and for a diluted sample

$$(3)\ T = Q(Cq) = Q(0) \times E^{Cq} \times d$$

where d is 1/(dilution factor). Isolating for Cq,

$$(4)\ Cq = -\frac{\log(d)}{\log(E)} + \frac{\log\left(\frac{T}{Q(0)}\right)}{\log(E)}$$

Equation 4 indicates that the semi-log plot Cq vs. log(d), also known as the standard curve plot, has a slope of -1/log(E), from which E can be determined and used to estimate initial quantities, Q(0), from test samples via Equation 1. Assuming that E is constant across all samples, all standard curves can be simultaneously fit with the constraint of slope equality, resulting in a globally estimated E. This is done in repDilPCR by using the **`lm`** function from the **`stats`** package in R. The fit has [(dilution points − 1) × (number of samples) − 1] degrees of freedom.

Assuming that for each sample the dilutions (d) and threshold (T) quantities used in Cq evaluation are identical for both a gene of interest and a reference gene, cancelling out d and taking the log of the right side in Equation 3,

$$(5)\ \log[Q_r(0)] + Cq_r \times \log(E_r) = \log[Q_g(0)] + Cq_g \times \log(E_g)$$

where “r” is the reference gene and “g” is the gene of interest. Isolating for Cq_g,

$$(6)\ Cq_g = \frac{\log(E_r)}{\log(E_g)} \times Cq_r - \frac{\log\left[\frac{Q_g(0)}{Q_r(0)}\right]}{\log(E_g)}$$

With both PCR efficiencies (E’s) and initial quantities (Q(0)) as constant, Equation 6 describes a linear relation between the two Cq values. Therefore, a Cq_g vs. Cq_r plot is a line with a y-intercept that indirectly corresponds to $Q_g(0)/Q_r(0)$, the expression ratio between the gene of interest and the reference gene. An increase of the ratio results in a downward shift of the line, whereas a decrease of the ratio results in an upward shift. The ratio can be quantified by using Equation 6 and an estimate of the PCR efficiency of the gene of interest (E_g) from standard curves. Since the slope of the Cq-Cq plots is

proportional to the ratio of the logarithms of the two PCR efficiencies that are taken as constant, the data are best fit by constraining the slopes similar to the standard curve fitting described above. Again, this is done in repDilPCR by using the **`lm`** function from the **`stats`** package.

2 Installation

repDilPCR can be installed on a local computer or on a server (see below). Alternatively, users can freely access and use a working installation hosted on a server at the German Cancer Research Center (DKFZ), Heidelberg, Germany (https://repdilpcr.eu). This service is anonymous, does not require registration and complies with common standards for protection of user data: raw data uploaded by the user are processed on the server and used to generate results that can be downloaded by the user; after the user closes the session by closing the browser window all uploaded data and processed results are automatically deleted from the server **(Warning: if you use a local installation of repDilPCR, do not store your data in the folder where repDilPCR is installed!)**.

2.1 Prerequisites for installation

- A working installation of R (version 3.6.0 or more recent) on a computer with a Linux or Windows operating system. (Theoretically installation on MacOS should be possible, too, but this has not been tested.) The RStudio integrated development environment is recommended for convenient use of the script but not required.
- The following R packages have to be installed: **`car, gridExtra, tidyverse, mice, PMCMRplus, ggbeeswarm`** and **`ggsignif`** (needed for both the ordinary R script and the Shiny app), as well as **`shiny, shinycssloaders`** and **`shinyalert`** (needed for the Shiny app only). It's possible that **`PMCMRplus`** will initially fail to install on a Linux system. The solution is to first install the GNU Multiple Precision Arithmetic Library (e.g. **`gmp-6.2.1.tar.lz`**) from https://gmplib.org/ and the GNU Multiple Precision Floating-Point Reliable Library (**`sudo apt install libmpfr-dev`** on a Debian-based distribution or **`sudo yum install mpfr-devel`** on a RedHat-based distribution).

2.2 Installation on a local computer

Download the zip archive of all files in the repository by clicking on "Code" and then on "Download ZIP" on the GitHub page of the repDilPCR project or by following this direct download link: https://github.com/deyanyosifov/repDilPCR/archive/refs/heads/main.zip. Unzip the archive, this action will create a new directory named **`repDilPCR-main`** in the current directory. You can rename the new directory to **`repDilPCR`** or whatever other name you choose and place it in a convenient place on your computer. For the purpose of this manual, we will assume that your installation is located in the directory **`repDilPCR`** in your home folder on a Linux machine, i.e. **`~/repDilPCR`**. If your situation is different, just replace the **`~/repDilPCR`** part in the further instructions with the actual path to your installation.

2.3 Installation on a server

This option is only possible on a server running Linux. Apart from the prerequisites stated above, you will need to install the Shiny Server. (It can be downloaded from https://www.rstudio.com/products/shiny/download-server/, detailed installation instructions are available at https://docs.rstudio.com/shiny-server/#install-shiny.) Installation of repDilPCR on a server is similar to installing on a local computer but the **`repDilPCR`** directory will have to be placed in **`/srv/shiny-server/`**. The shiny user must have read and write access to **`/srv/shiny-server/repDilPCR`** and its contents.

3 Usage

repDilPCR can be used both as an ordinary R script on a local computer or as a Shiny app (either on a local computer or on a server) accessed through a web browser. The workflow is summarized in Fig. S1.

3.1 Preparation of input data

This preparatory step is the same no matter whether you intend to use the R script or the Shiny app. Preparing the data will take you some time, especially the first time when you do it, but don't worry, what might seem like wasted time is actually very well invested time because it will bring you considerable time savings later on by enabling automated analysis by repDilPCR.

The input data have to be arranged in a CSV file following a specific format which is different depending on whether one wants to feed into the program unprocessed Cq values obtained from an experiment performed according to the dilution-replicate approach or already calculated relative expression values. Exemplary input data tables for these two use cases are provided in the files **`Test_data.csv`** and **`Test_data_precalc.csv`**, respectively, which are available in the installation directory or can be downloaded using the buttons on the `About/Help` tab of the repDilPCR program. In the exemplary files, points are used as decimal separators and commas as field separators (to separate values in each row). It is also possible to use commas as decimal separators and semicolons as field separators – the default regional setting in most European countries. The program will recognize the format automatically. Cq values can usually easily be exported by the software controlling your qPCR machine. An important thing that you have to pay attention to is that you **select a common threshold for all amplicons** in your experiment before exporting the Cq values. Depending on the manufacturer of the machine and the respective software, Cq values might be referred to as Ct ("cycle threshold") or Cp ("crossing point") values but these different names stand for the same thing. Here, we adhere to the MIQE guidelines and the respective terminology (Cq = quantification cycle) (Bustin, et al., 2009).

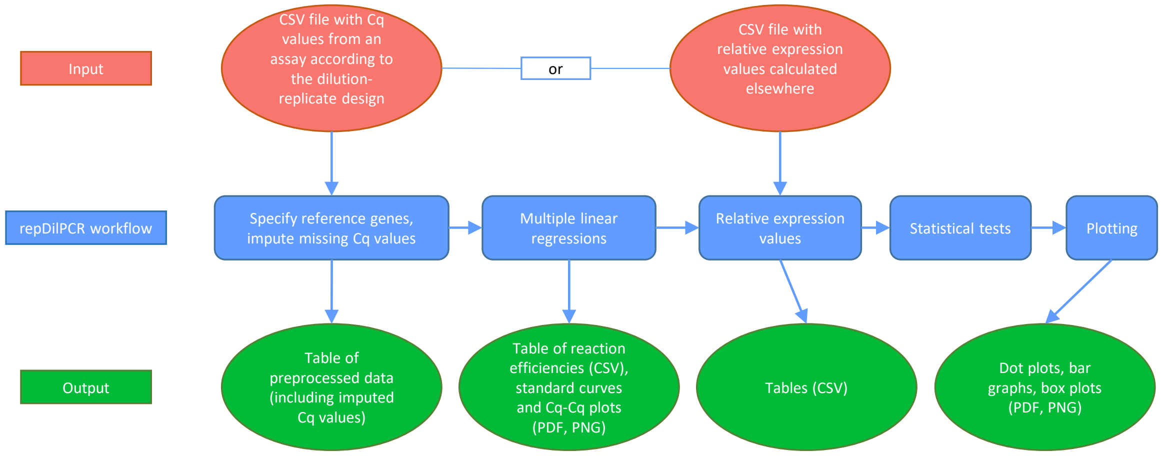

Figure S1: repDilPCR workflow.

In the case of using Cq values, the CSV file needs to have the following layout: The first row contains column titles. The first three columns have predetermined names that must not be changed. The first column is called "Replicates" and it should contain the names of the samples with a suffix that identifies the biological replicate. The suffix consists of an underscore ("_") plus additional numbers and/or letters. For example, if you have an experimental group called "HeLa" and your experiment contained biological triplicates, they could be entered in the column as "HeLa_1", "HeLa_2" and "HeLa_3". The same convention can be used if your experiment compares groups of patients and each patient provided

a single sample. In this case, each subject would be considered a biological replicate of the respective group and assigned an individual suffix number, e.g. if you compared the expression of a gene in 3 groups of 10 subjects each - healthy, leukemia and lymphoma, your labels in the "Replicates" column might be "Healthy_1", "Healthy_2", ..., "Healthy_10", "Leukemia_1", "Leukemia_2", ..., "Lymphoma_1", "Lymphoma_2" and so on (or you could use subject name initials, instead of numbers, as long as they don't occur more than once). If your experiment did not include replicates, the single sample can be entered as either "HeLa_1" or just "HeLa" in the table, it doesn't matter. The underscore character is reserved for the purpose of identifying replicates and should not occur elsewhere in the names of the samples. The second column is called "Pairs" and can contain optional information about grouping of samples in pairs. This is useful if the experiment consists of samples or sample groups that should be compared and statistically evaluated in pairs, e.g. if you have a number of cell lines in each of which you have measured the expression of your gene of interest in two conditions (control and treated) and you are interested in comparing these two conditions for each cell line but don't care about comparisons among cell lines. Then, all replicates from both samples of a given cell line should be assigned the same number in the column "Pairs". For example, all replicates (biological as well as dilution replicates) of "HeLa control" and "HeLa treated" should receive a number 1 in the "Pairs" column, all replicates of "Jurkat control" and "Jurkat treated" should receive a number 2 and so on. In this way, the program will know which samples constitute a pair. If your experiment does not contain pairs or you do not wish to use this functionality, leave the column empty but do not delete its title. The third column is called "Dilution" and contains the dilution factors according to the dilution-replicate design. For example, if you had 5-fold serial dilutions in your experiment, you can use as factors the numbers 1, 5 and 25 (the actual numbers do not matter, only the ratios among them are important, so you could just as well use 2, 10 and 50). If we return to the example given above, this means that for each biological replicate "HeLa_1", "HeLa_2" and "HeLa_3" you would have three dilution replicates, accordingly each biological replicate should be listed three times in the "Replicates" column and the respective rows in the "Dilution" column should be filled with the numbers 1, 5 and 25 where 1 corresponds to the most concentrated dilution replicate and 25 corresponds to the most diluted one. The following columns should contain the Cq values for the assessed genes, first the reference genes (RG) and then the genes of interest (GOI). The titles of these columns should be the names of the respective genes/amplicons. Here is a quick look at a part of the Test_data.csv file as an example of the required format:

```
Replicates,Pairs,Dilution,RG1,RG2,RG3,GOI1,GOI2
CellLine1_1,1,1,22.91,24.69,21.85,23.06,22.31
CellLine1_1,1,5,25.67,27.97,23.99,25.68,24.25
CellLine1_1,1,25,28.08,30.71,26.66,28.42,27.28
CellLine1_2,1,1,23.70,25.33,21.70,23.66,21.55
CellLine1_2,1,5,26.28,27.96,23.63,26.13,24.92
CellLine1_2,1,25,27.74,30.05,25.90,28.02,26.48
CellLine1_3,1,1,23.60,24.67,21.82,23.21,21.55
CellLine1_3,1,5,26.14,28.69,24.29,25.82,24.17
CellLine1_3,1,25,27.42,30.23,25.94,27.64,26.70
.............................................
CellLine1_9,1,1,22.86,24.96,21.44,22.04,21.34
CellLine1_9,1,5,25.45,27.97,23.81,25.49,25.08
CellLine1_9,1,25,27.48,30.03,26.40,28.16,26.29
CellLine1 (trt)_1,1,1,23.27,24.66,21.50,25.15,23.48
CellLine1 (trt)_1,1,5,25.60,27.38,24.32,26.98,25.74
CellLine1 (trt)_1,1,25,28.09,30.45,26.09,28.95,28.12
...................................................
CellLine1 (trt)_9,1,1,22.35,25.30,21.46,24.59,22.49
CellLine1 (trt)_9,1,5,25.58,27.69,24.27,26.67,25.71
CellLine1 (trt)_9,1,25,27.57,29.78,26.16,29.12,27.54
CellLine2_1,2,1,23.41,22.73,22.28,23.43,21.03
CellLine2_1,2,5,25.72,26.29,24.86,26.31,24.36
CellLine2_1,2,25,28.06,27.88,26.61,27.54,26.05
```

```
CellLine2_2,2,1,22.99,23.16,21.27,23.16,21.49
.............................................
```

In this example fragment, three experimental groups are present ("CellLine1", "CellLine1 (trt)" and "CellLine2"), the first two of which form a pair (baseline vs. treated), as the same number (1) is present in the second column of all rows occupied by replicates of these two samples ("CellLine2" is part of another pair, as its replicates have the number 2 in the second column.) Each experimental group has 9 biological replicates and each of them has 3 dilution replicates (denoted by the dilution factors 1, 5 and 25 in the third column). Note that they are not called "technical replicates" as the concentration of the template in them is not identical, nevertheless they simultaneously fulfil two roles: producing standard curves and controlling technical variance. The example fragment above includes three reference genes (labelled "RG1", "RG2" and "RG3") and two genes of interest ("GOI1" and "GOI2"). The order in which you enter your samples in the table is important because this will also be the order in which your samples will be displayed on the graphs, i.e. the sample that the program finds in the first row will become the leftmost sample on the graphs and so on.

In the case of using relative expression values, the CSV file that has to be prepared has a simpler layout. Again, the first row contains column titles but now only the first two columns are obligatory and with predetermined names that must not be changed: "Replicates" and "Pairs". Their specification is the same as in the case when Cq values are used (see above). The next columns should contain the relative expression levels (linearly scaled) of the evaluated genes of interest in each biological replicate. Accordingly, the titles of these columns would be the respective gene/amplicon names. Here is a quick look at a part of the `Test_data_precalc.csv` file as an example of the required format:

```
Replicates,Pairs,GOI1,GOI2
CellLine1_1,1,1.58,1.42
CellLine1_2,1,1.33,1.70
CellLine1_3,1,1.81,1.98
.......................
CellLine1_9,1,1.91,1.56
CellLine1 (trt)_1,1,0.58,0.60
.............................
CellLine1 (trt)_9,1,0.63,0.78
CellLine2_1,2,1.10,1.72
CellLine2_2,2,1.19,1.27
.......................
```

In this example fragment, three experimental groups are present ("CellLine1", "CellLine1 (trt)" and "CellLine2"), the first two of which form a pair (baseline vs. treated), as the same number (1) is present in the second column of all rows occupied by replicates of these two samples ("CellLine2" is part of another pair, as its replicates have the number 2 in the second column.) Each experimental group has 9 biological replicates. The example fragment above includes two genes of interest ("GOI1" and "GOI2"). The order in which you enter your samples in the table is important because this will also be the order in which your samples will be displayed on the graphs, i.e. the sample that the program finds in the first row will become the leftmost sample on the graphs and so on.

3.2 Usage of the Shiny app

The Shiny app can be used via any modern web browser. Users have two options:

- access a publicly available Shiny server with repDilPCR installed on it, for example the installation hosted at the German Cancer Research Center (DKFZ) in Heidelberg (https://repdilpcr.eu)

- if repDilPCR is installed on their local computer (see above for how-to), they can start the R environment and issue the following commands:

```
library(shiny)
runApp("~/repDilPCR/app.R", launch.browser = TRUE)
```

  replacing the `~/repDilPCR` part with the actual path to their installation, if deviating. This should launch the program and automatically start a new browser window or tab to access it.

The workflow is as follows:

3.2.1 Upload your data

This is straightforward: just click the Browse button, select your already prepared CSV file (see above) and upload it. At this point, if your file was formatted correctly, your data should appear in the pane to the right of the app control panel. The program will automatically know whether your data contain Cq values or already calculated relative expression levels. If the browser window grays out, this means that an error has occurred, most probably because your file was not formatted correctly. You will have to refresh the page and upload a corrected version of your CSV file that adheres to the specifications stated above.

3.2.2 Select your reference genes and (optionally) impute missing Cq values

(This whole step is only relevant when working with Cq values.)

This is very easy because you have already arranged the columns with the Cq values of your reference genes in the place where the program expects them to be: between the column "Dilution" and the columns with the Cq values of your genes of interest. The only thing that the program does not yet know is how many of them there are. You provide this information by entering the correct number under **`Number of reference genes`** in the app control panel.

In principle, if you have multiple reference genes in your experiment (strongly recommended, see this classical work: https://doi.org/10.1186/gb-2002-3-7-research0034 (Vandesompele, et al., 2002)), the Cq values from the separate reference genes are averaged to calculate the normalization factor for each biological replicate. With the dilution-replicate design this averaging has to be performed at each dilution level. In practice, this creates a single virtual reference gene for normalization with the following properties: its Cq value at each dilution step is the average of the Cq values of the included real reference genes, hence the apparent PCR efficiency of this virtual gene will be determined by the PCR efficiencies of the included reference genes.

As it happens every now and then, a single or several reactions on your PCR plate may fail (pipetting errors, contamination with inhibiting substances, etc.). Consequently, if one of the dilution replicates for one of the reference genes fails, the normalization factor for this dilution level of the affected biological replicate would not be able to be calculated although the respective dilution replicates for the other reference genes are perfectly fine. Furthermore, Cq values for the genes of interest would not be able to be plotted against missing normalization factor data points when constructing the Cq-Cq plots. This would be an annoying situation because it would lead to a bigger loss of information than actually inflicted by the failure of the single reaction. However, one can take advantage of the facts that (1) correlation should exist between the Cq values of the reference genes, (2) normally there would be other biological replicates with intact sets of dilution replicates across all reference genes and (3) Cq values depend on the dilution level. This information allows one to construct a model and perform imputation of the missing values and thus to minimize the uncertainty in determining the normalization factors. This is easily done in repDilPCR by selecting the respective option in the app control panel. This capability has to be used judiciously: although it works very reliably in the majority of cases, the power of imputation will come to a limit if your experiment has too many missing values or no biological

replicates. As a good practice, we suggest that when users present their results they should also report the number of missing data points that have been imputed. Details on the imputation method used by repDilPCR can be found in the chapter “Technical notes” of this Supplementary Information.

You can see the full preprocessed dataset (original and imputed values together in a single table) by clicking on the tab **Preprocessed input data** in the right pane of the program window (this tab is only visible when working with Cq values). This preprocessed table will contain two additional columns titled "Samples" and "NF", where "NF" is the virtual reference gene whose Cq values are obtained by averaging the Cq values of the real reference genes. If you want, you can download this table by going to **Download results / Intermediate data** and clicking on the **Download table with imputed Cq values (if any)** button.

3.2.3 Analyze your data

Simply click on the big green **Analyze** button in the app control panel. All optional parameters below the button can be adjusted afterwards, too. Processing your results will typically take a few seconds with possible deviations depending on the size and complexity of your dataset. When the analysis is finished, the program will take you to the **Results** tab in the right pane of the program window to get a quick look at the results (see point 3.2.5. for detailed description of data visualization options in the **Results** tab). However, if you started from Cq values, it is good practice to first review the goodness of fit of the regression plots (see point 3.2.4.).

3.2.4 Check the results of the regression analysis

(This step is only relevant when working with Cq values.)

It is good practice to inspect the standard curves and Cq-Cq plots before proceeding further to make sure that there are no outliers and that the coefficients of determination (R^2) are large enough (close to 1). For example, a sample contaminated with a PCR inhibitor might show unexpected behaviour as dilution would reduce the concentration of the inhibitor and thus dilution-replicates may exhibit steeper amplification curves than the undiluted sample. As a result, single data points may be far away from their respective fitted curve. Results from such samples should be interpreted with caution or excluded from the analysis.

Go to the **Regression plots** tab (only visible when working with Cq values) and two subtabs will appear: **Standard curves** and **Cq-Cq plots**. Note that depending on the number of your samples it may take some time (seconds) from the moment you click on one of them till the graphs are rendered and displayed.

The standard curves plots will be listed one under the other in the following order: first the genes of interest, then the virtual reference gene ("NF") produced by averaging the Cq values of the real reference genes and then the real reference genes themselves. Each plot will consist of as many standard curves as there are samples (biological replicates) and all of them will be parallel to each other (because we have performed multiple linear regression with parallel slopes). Different colour will be assigned to each sample (biological replicate) to help you identify them on the plots. Statistical summary will be displayed under the legend of each plot and will include residual standard error with degrees of freedom, number of missing observations (if any), coefficients of determination (R^2 and adjusted R^2), F-statistic with degrees of freedom and p-value of the regression. The PCR efficiency ("Eff") for each amplicon will be displayed in the respective plot together with its 95%-confidence interval. This is what a typical standard curves plot looks like:

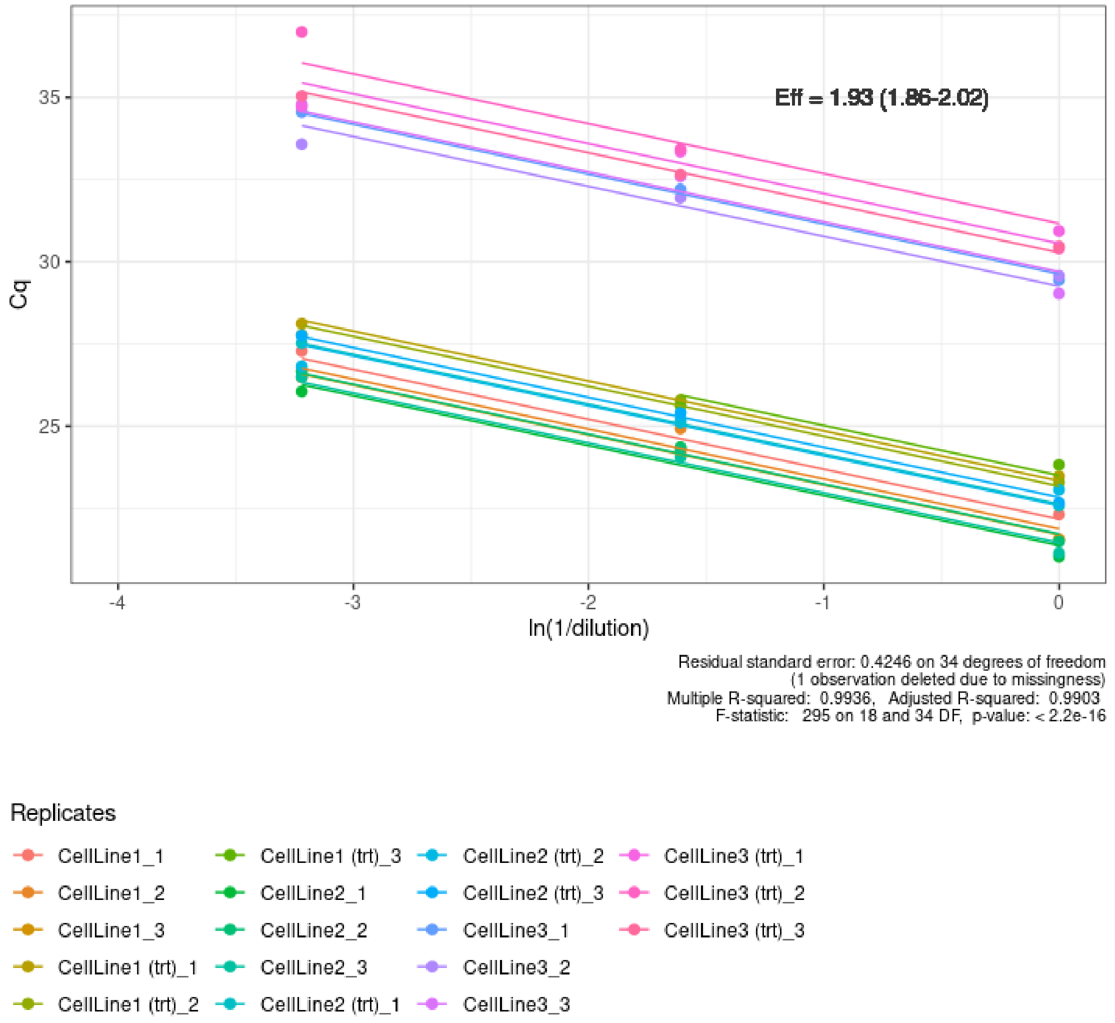

Figure S2: Example of standard curves. The plot was prepared using the exemplary data in the file Test_data.csv. For clarity, only (the first) three replicates per sample are shown.

Efficiencies can be downloaded as a table by navigating to **`Download results / Intermediate data`** and clicking on the **`Download table with calculated efficiencies`** button. All standard curve plots can be downloaded as a PDF file or as a ZIP archive of PNG files by clicking on the respective download button. The file format can be changed by selecting the respective option at the bottom of the app control panel. In the case of the PNG format, one can also select the desired width, height and resolution.

The **`Cq-Cq plots`** subtab will include Cq-Cq plots for each gene of interest against the virtual reference gene. The y-intercepts of the regression lines on these plots are used in a next step to calculate the relative expression of genes of interest in the different samples. Again, each plot will consist of as many parallel regression lines as there are samples (biological replicates) and will have a statistical summary underneath, as for the standard curve plots. Similarly, Cq-Cq plots can be downloaded as a file from the **`Download results / Intermediate data`** subtab. This is what a typical Cq-Cq plot looks like:

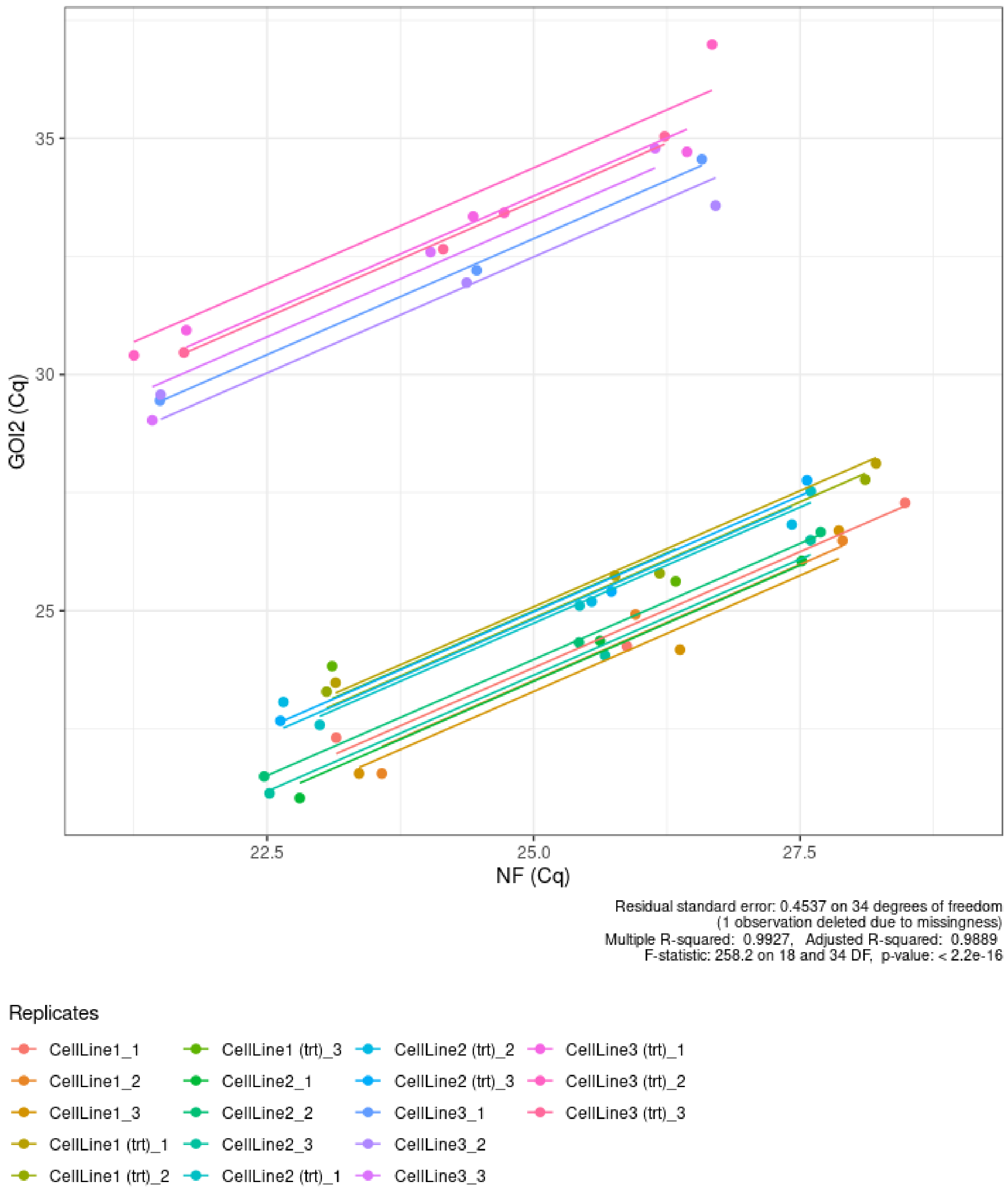

Figure S3: Example of a Cq-Cq plot. The plot was prepared using the exemplary data in the file Test_data.csv. For clarity, only (the first) three replicates per sample are shown.

3.2.5 Visualize the results

The **Results** tab has two subtabs: **Plots in linear scale** and **Plots in logarithmic scale**. Each of them will have its own subtabs, that will display your results as different types of graphs. The exact types of plots that are available will depend on the type of statistical tests that you have chosen on the left: parametric or non-parametric. If "parametric" is selected, the available choices will be: **Dot plots (all points), Dot plots (means and standard deviations)** and **Bar graphs (means and standard deviations)**. If "non-parametric" is selected, the available choices will be **Dot plots (all points)** and **Box plots**. The control panel on the left gives you the opportunity to change the visual appearance of the plots, as well as to control parameters of the statistical tests. Labelling of samples and genes will happen automatically based on the information in the uploaded data table and will not require additional effort from you.

3.2.6 Perform statistical tests

repDilPCR aims to make the process of testing statistical hypotheses easy even for users without much knowledge of statistics by automatically selecting appropriate statistical tests depending on the context and properties of the data. (If you don't want to perform statistical tests, remove the tick from the checkbox **Test for statistically significant differences between samples or experimental groups** in the control panel). The user can choose the broad type of statistical test (**parametric** or **non-parametric**) and the comparisons to be tested for statistically significant differences (**all to one (all to reference), all pairs** and **selected pairs**) by clicking on the respective radio buttons in the control panel. The significance level (α) can also be freely selected. To make usage of parametric tests possible, all statistical tests are performed on logarithmically transformed data, even when the user chooses to display plots in linear scale (qPCR data are not normally distributed on a linear scale! (Rieu and Powers, 2009)).

When the option **all to one (all to reference)** is selected, one of the experimental groups (by default the first one in the data table, resp. the leftmost on the plot) will be compared against all other experimental groups (examples in Fig. S4-S6). Any other experimental group can be made the reference by entering its name in the text box under the option **Reference sample or experimental group** in the control panel and pressing the **Analyze** button again. Experimental groups in which the expression of a given gene of interest is significantly different from that in the reference group will be marked as such with asterisks or with p-values based on the user's choice. The statistical test that was automatically chosen and performed in each particular case will be listed below the respective plot. Read the chapter “Technical notes” below for more details on the logic that selects the statistical test.

When the option **all pairs** is selected, each of the experimental groups will be compared against any other experimental group (example in Fig. S7). Comparisons for which the expression of a given gene of interest is significantly different between the groups will be marked by significance bars with either p-values or asterisks on top of them depending on the user's choice. The statistical test that was automatically chosen and performed in each particular case will be listed below the respective plot. Read the chapter “Technical notes” below for more details on the logic that selects the statistical test.

When the option **selected pairs** is selected, experimental groups will be compared in pairs according to the pairing information provided in column "Pairs" of the data table (see “*Preparation of input data”* above and Fig. S8). Comparisons for which the expression of a given gene of interest is significantly different between the groups constituting a pair will be marked by significance bars with either p-values or asterisks on top of them depending on the user's choice. The statistical test that was automatically chosen and performed in each particular case will be listed below the respective plot. Read the chapter “Technical notes” below for more details on the algorithm that selects the statistical test.

These are some exemplary plots:

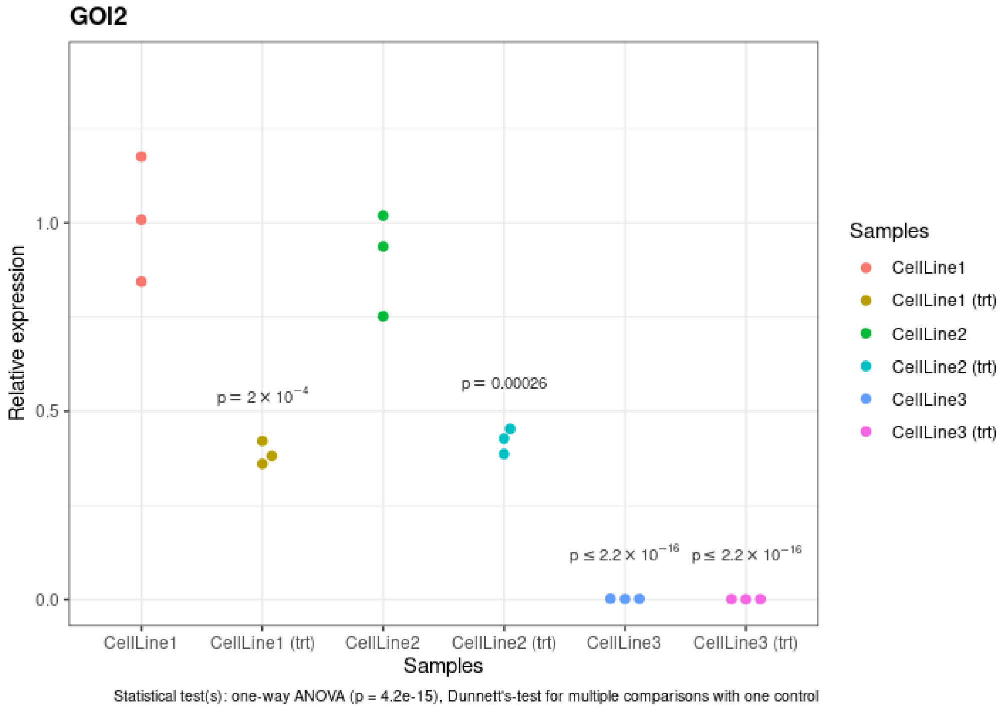

Figure S4: Dot plot (all points) in linear scale, all groups compared to a referent one. The plot was prepared using the exemplary data in the file Test_data.csv. Only (the first) three replicates per sample are shown, as in the plots above.

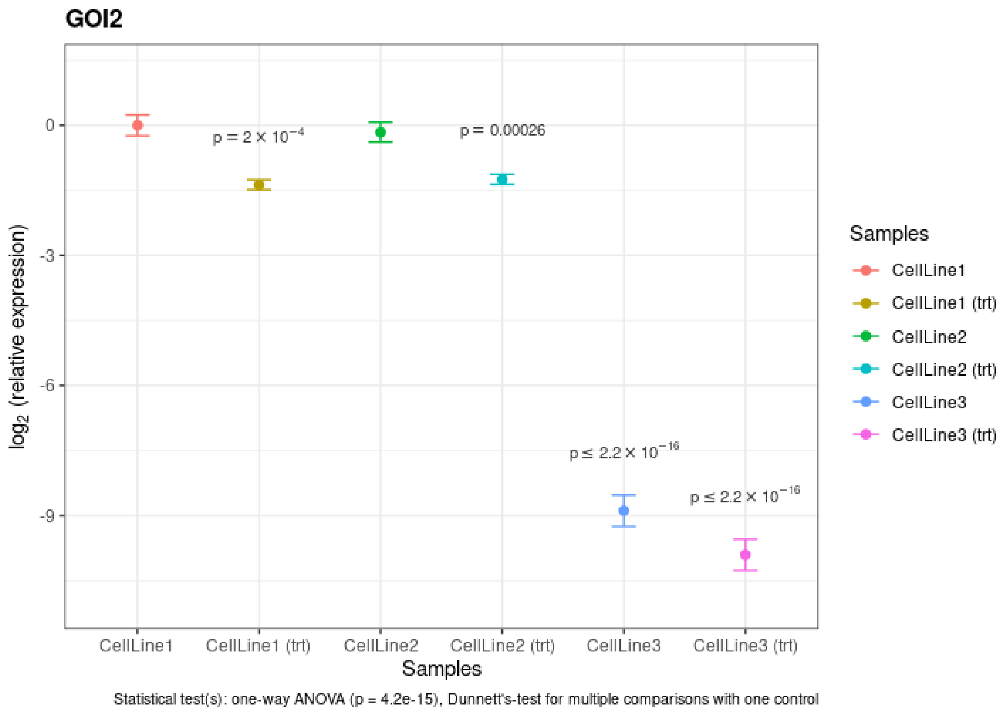

Figure S5: Dot plot (means and confidence intervals) in logarithmic scale, all groups compared to a referent one. The plot was prepared using the exemplary data in the file Test_data.csv. Only (the first) three replicates per sample are shown, as in the plots above.

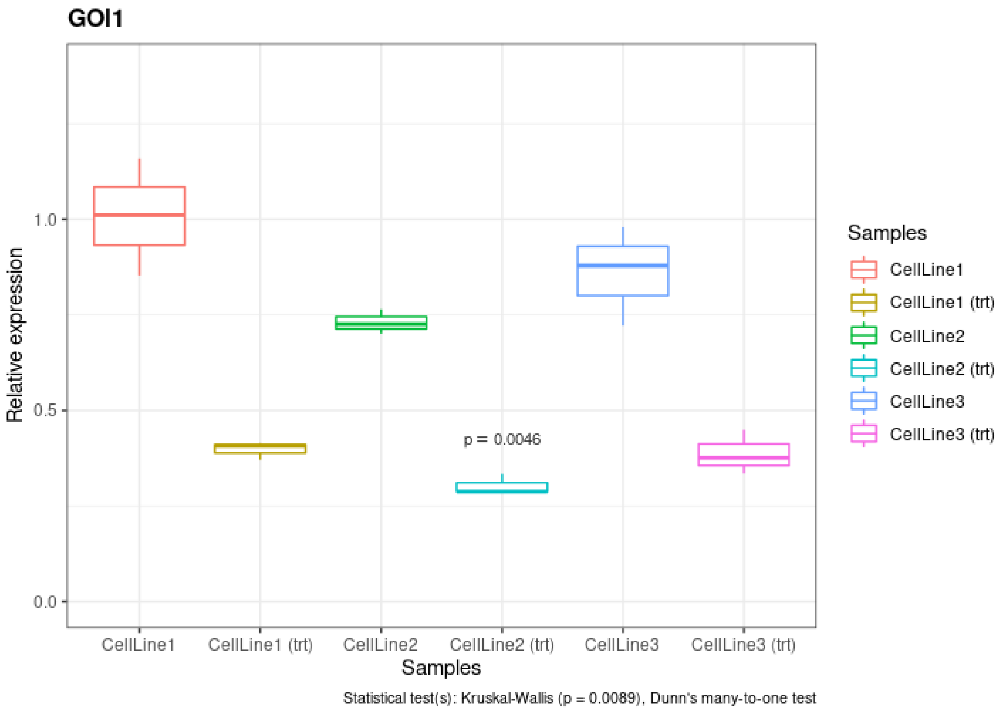

Figure S6: Box plot in linear scale, all groups compared to a referent one via non-parametric tests. The plot was prepared using the exemplary data in the file Test_data.csv. Only (the first) three replicates per sample are shown, as in the plots above.

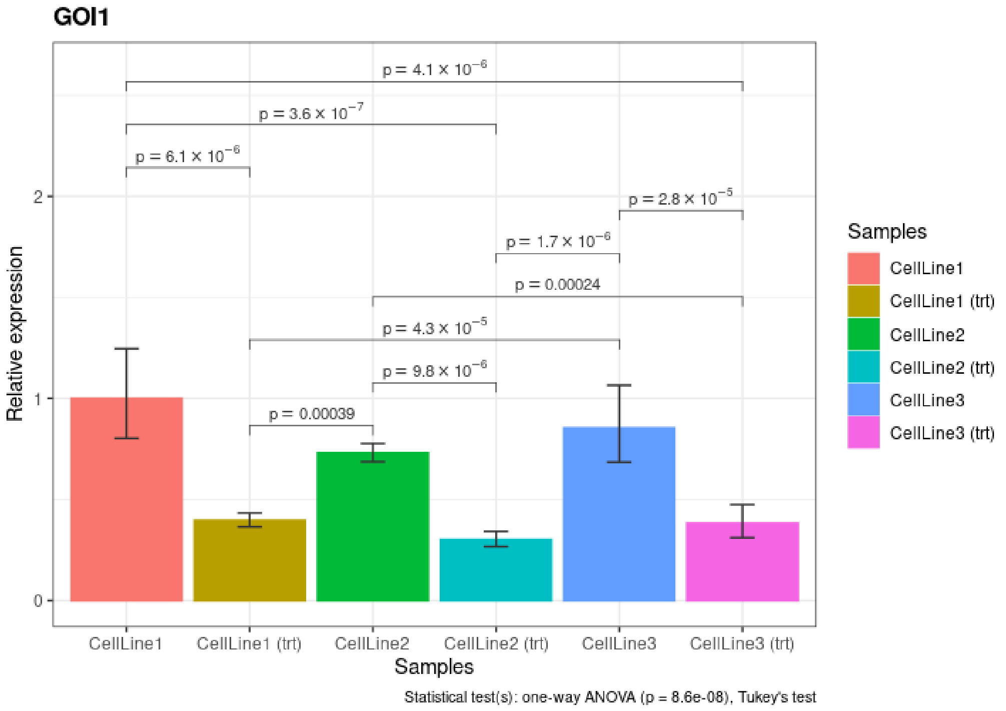

Figure S7: Bar graph in linear scale, all groups compared to one another. The plot was prepared using the exemplary data in the file Test_data.csv. Only (the first) three replicates per sample were used, as in the plots above.

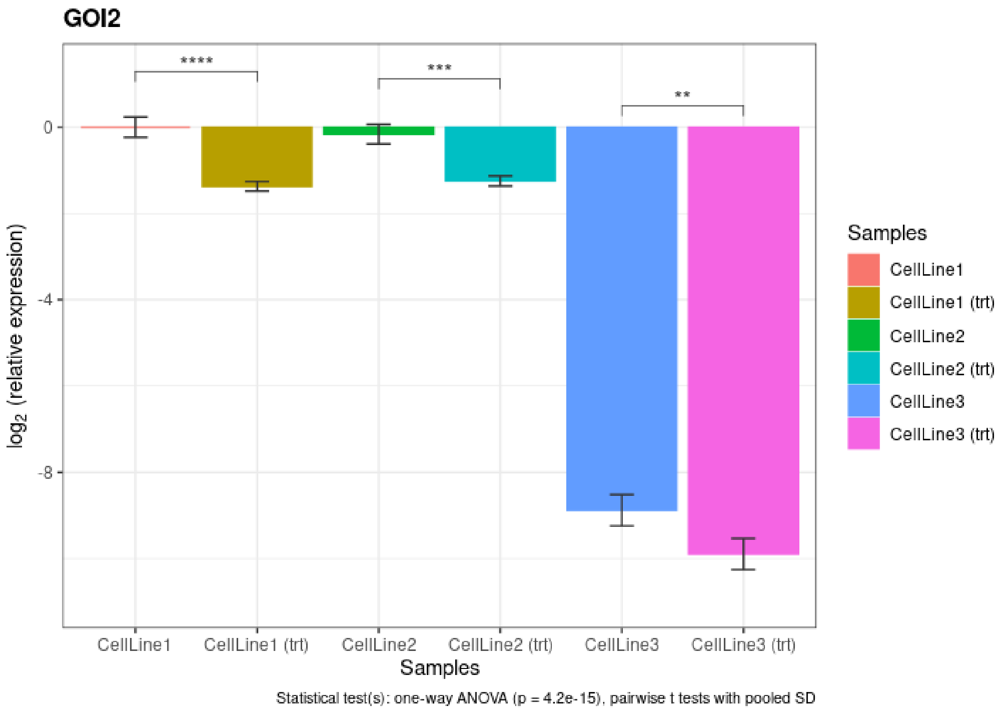

Figure S8: Bar graph in logarithmic scale, groups within selected pairs compared to one another. The plot was prepared using the exemplary data in the file Test_data.csv. Only (the first) three replicates per sample were used, as in the plots above.

3.2.7 Adjust the visual appearance of the plots to your needs

Users can adjust the font size on plots by using the respective input box **`Font size of text on plots`** in the control panel. Changing the preset number (9) will scale proportionately all different font sizes that occur on plots.

Statistical significance can be denoted on plots with either numeric p-values or asterisks. The choice can be stated by selecting the respective radio button under **`Display format of statistical significance on plots`**. In the case of asterisks, their number corresponds to the significance level according to the following convention: * denotes $0.01 < p \leq 0.05$, ** denotes $0.001 < p \leq 0.01$, *** denotes $0.0001 < p \leq 0.001$, **** denotes $p \leq 0.0001$.

In the case of statistical tests comparing all possible pairs of experimental groups, the resulting plots might become overcrowded with significance bars. repDilPCR strives to distribute them in a way that they don't overlap each other and its algorithm works well in most cases. However, if there are a lot of experimental groups and a lot of the comparisons are significantly different, the automatic algorithm may fail to prevent overlapping of significance bars. Users can influence the algorithm by changing the spacing factor under **`Distance between significance bars on plots`**. Its default value is 1.5. Increasing this heuristic parameter will increase the distance between significance bars. Conversely, if the distances between significance bars are too big and they are wasting space on plots, you can try decreasing the spacing factor.

3.2.8 Download results

All plots and tables that repDilPCR produces can be downloaded from the **`Download results`** tab. It has three subtabs: **`Plots`**, **`Tables`** and **`Intermediate data`**.

The **`Plots`** subtab contains download buttons for plots of final results. They are organized into two subtabs: **`Plots in linear scale`** and **`Plots in logarithmic scale`**. Each of them will contain download buttons for different types of graphs: dot plots (all points), dot plots (means and confidence intervals), bar graphs and box plots. The exact types that will be available depend on the choice of statistical tests from the control panel (see 3.2.6.). All plots can be downloaded in the PDF or PNG file format. The available formats can be chosen by clicking on the respective radio button under **`Format of graphical output (only for downloadable files)`** in the control panel. PDF files will be multi-page, meaning that the plots for all genes of interest will be put together in a single file on separate pages. Each PNG file will contain a single plot (gene) but all plots of a particular type will be grouped together and downloaded as a ZIP archive. In all cases, downloaded files will have automatically created informative file names that will include the name of the dataset (uploaded data file) and the plot type. Additionally, plots in logarithmic scale will have "log" in their file names. Plots in PDF files are stored as vector graphics and they are scalable without loss of resolution. PNG files use raster graphics and their resolution depends on the size of the image and the number of pixels. Users can specify these parameters in the control panel to get a PNG file that will conform to their requirements (or the requirements of a journal in which they intend to publish).

The **`Tables`** subtab enables downloading of the final results as tables in CSV format. Four choices are possible: "relative expression values for each replicate (linear scale)", "relative expression values for each replicate (logarithmic scale)", "mean expression values (linear scale)" and "mean expression values (logarithmic scale)".

The **`Intermediate data`** subtab enables downloading of tables and plots created during the intermediate steps of the analysis: table of preprocessed input data including imputed Cq values (if any), table of calculated efficiencies for the different amplicons, plots of standard curves and Cq-Cq plots. The tables are in CSV format and the plots can be downloaded in PDF or PNG format depending on the selected settings (see above).

3.3 Usage of the R script

The R script (repDilPCR.R) is an alternative of the Shiny app and offers the same functions but might be preferred by users with experience in R. It offers the advantage of even faster processing of data as the user just has to specify the path to the data, set the preferences for the analysis and then execute the script. Then all intermediate and final results will be automatically saved in the same directory as the raw data without the need to click around in a graphical interface and to download result files one by one.

The executable script is called repDilPCR.R and is located in the directory where repDilPCR has been installed. It depends on repDilPCR_lib.R which contains the actual functions and has to be in the same directory as repDilPCR.R. Before executing, the user should open repDilPCR.R in a text editor or more conveniently in RStudio and modify the variables as needed for the particular analysis. All user-modifiable variables can be found in the beginning of the file (lines 10-27) right after the header with the basic information about the script and are separated into two parts: mandatory variables and optional variables. All variables are the same as those found in the graphical interface of the Shiny app (see the previous section for more detailed explanation of their meaning and usage).

The mandatory variables include the path to the dataset that is to be processed and the number of reference genes that it contains. They are called **`input.table`** and **`RG`**, respectively. The script won't

run or will run erroneously if you forget to set these variables to reflect the actual situation with your data.

input.table - enter the full path to the input CSV file between the quotation marks.

RG - corresponds to **Number of reference genes** in the graphical interface (see point 3.2.2. in "Usage of the Shiny app"). Default value: **3**. Change it as needed.

The optional variables are:

impute - corresponds to **Impute missing Cq values of reference genes** in the graphical interface (see point 3.2.2. in "Usage of the Shiny app"). Set to **TRUE** (default) or **FALSE** to respectively enable or disable imputation of missing Cq values of reference genes. Do not set to **TRUE** if your experiment does not contain replicates.

ref.sample - corresponds to **Reference sample or experimental group** in the graphical interface. It specifies the reference sample in which gene expression will be regarded as 1 (100%) on linear scale and 0 on log2-scale, respectively. If set to **"default"** (default), this will be the first sample in the table, resp. the leftmost sample on the plots. Change to the name of another sample (without a trailing underscore and replicate number) to make it the reference sample. If the variable is set to the empty string **""**, results will be shown in their original form, without forcing any particular sample to be 1 (100%) or 0.

statistics - corresponds to **Test for statistically significant differences between samples or experimental groups** in the graphical interface. Set to **TRUE** (default) or **FALSE** to respectively enable or disable tests for statistical significance between samples or experimental groups.

test.type - corresponds to **Type of statistical test(s)** in the graphical interface (see point 3.2.6. in "Usage of the Shiny app"). Possible values: **"parametric"** (default) and **"non-parametric"**.

posthoc - corresponds to **Comparisons to test for statistically significant differences** in the graphical interface (see point 3.2.6. in "Usage of the Shiny app"). It specifies the comparisons to be tested for statistically significant differences. Possible values: **"all to one"** (default), **"all pairs"** and **"selected pairs"**.

alpha - corresponds to **Significance level (α)** in the graphical interface (see point 3.2.6. in "Usage of the Shiny app"). Default value: **0.05**.

font.size - corresponds to **Font size of text on plots** in the graphical interface (see point 3.2.7. in "Usage of the Shiny app"). Default value: **9**.

sign.repr - corresponds to **Display format of statistical significance on plots** in the graphical interface (see point 3.2.7. in "Usage of the Shiny app"). Possible values: **"values"** (default) and **"asterisks"**.

sp.f - corresponds to **Distance between significance bars on plots** in the graphical interface (see point 3.2.7. in "Usage of the Shiny app"). Default value: **1.5**.

plot.format - corresponds to **Format of graphical output (only for downloadable files)** in the graphical interface (see point 3.2.6. in "Usage of the Shiny app"). Possible values are **"PDF"** (default), **"PNG"**, **"both"** and **"none"**.

png.size - corresponds to width and height in **Settings for PNG plots** in the graphical interface (see point 3.2.6. in "Usage of the Shiny app"). The default value is **"c(190,134)"** (interpreted as 190 mm width and 134 mm height) and fits well to the A5 page size format in landscape orientation.

png.dpi - corresponds to **Resolution in dpi** in the graphical interface (see point 3.2.6. in "Usage of the Shiny app"). The default value is **96**.

After changing the variables as necessary, save the modified file and start the script. There are different ways to do this:

- You can navigate in the terminal to the directory where repDilPCR has been installed and then issue the command

  ```
  Rscript repDilPCR.R
  ```

- If you have the file repDilPCR.R open in RStudio, all you have to do is to select all text (**Ctrl+A**) and then click on **Run**.

- You can start R in a terminal and then enter the command

  ```
  source("~/repDilPCR/repDilPCR.R")
  ```
  (if repDilPCR is installed in a different directory on your computer, you will have to modify the path accordingly).

It will take from a few seconds up to a minute for the script to execute depending on the speed of your computer, the size of the dataset, the selected parameters of the analysis and eventually the presence of missing Cq values of the reference genes. All results will be delivered in the directory containing the input dataset as long as the variable **input.table** has been set corrrectly.

4 Technical notes

4.1 Imputation of missing Cq values

Imputation in repDilPCR is performed using the R package **mice** with the options **method = 'midastouch', m = 20, maxit = 20**, i.e. the imputation method that is used is weighted predictive mean matching and 20 multiple imputations are performed, each of them with 20 iterations. The mean of the 20 imputed values is taken as the final result and passed further on. In principle, mice depends on a random number generator to perform its calculations, which can lead to slightly different results when the same data is analyzed multiple times. In order to make repDilPCR return reproducible results when the same dataset is analyzed multiple times, the random number generator state is kept constant by using a hard-coded seed **set.seed(40075017)**.

4.2 Algorithm for choosing statistical tests

When the options **all to one (all to reference)** and **parametric** are selected, repDilPCR will first evaluate the number of experimental groups and whether variance is homogenous among them by using Levene's test (the function **leveneTest** from the package **car**). If there are only two groups, an unpaired two-sided t-test will be performed. If Levene's test was not significant ($p > 0.05$), the variances of the two groups will be treated as equal and the pooled variance will be used by the t-test. If Levene's test was significant ($p \leq 0.05$), Welch's approximation will be used. If there are more than two groups, ANOVA (analysis of variance) will be performed using the function **aov** if Levene's test was not significant or Welch's ANOVA using the function **oneway.test** will be performed in case Levene's test was significant (both ANOVA functions are from the package **stats**). If the p-value of the ANOVA

test is lower than or equal to the chosen significance level (α, 0.05 by default), repDilPCR will perform a post-hoc test: Dunnett's multiple comparisons test with one control (if Levene's test was not significant) or Tamhane-Dunnett's multiple comparisons test with one control (if Levene's test was significant). The last two tests are performed by the functions **dunnettTest** and **tamhaneDunnettTest** from the package **PMCMRplus**, respectively. Post-hoc tests automatically take care of the multiple testing problem, so reported p-values don't need adjustment when considered at the level of a single plot (single gene of interest). If you have evaluated many genes of interest, you might need additional correction for multiple testing depending on your research question.

When the options **all to one (all to reference)** and **non-parametric** are selected, repDilPCR will first evaluate the number of experimental groups. If there are only two groups, an unpaired two-sided Wilcoxon test (also known as Mann-Whitney test) will be performed using the function **wilcox.test** from the package **stats**. If there are more than two groups, a Kruskal-Wallis rank sum test will be performed using the function **kruskalTest** from the package **PMCMRplus**. If the p-value of the Kruskal-Wallis test is lower than or equal to the chosen significance level (α, 0.05 by default), repDilPCR will perform Dunn's non-parametric many-to-one comparison test as a post-hoc test using the function **kwManyOneDunnTest** from the package **PMCMRplus**. Post-hoc tests automatically take care of the multiple testing problem, so reported p-values don't need adjustment when considered at the level of a single plot (single gene of interest). If you have evaluated many genes of interest, you might need additional correction for multiple testing depending on your research question.

When the options **all pairs** and **parametric** are selected, repDilPCR will first evaluate whether variance among experimental groups is homogenous by using Levene's test (the function **leveneTest** from the package **car**) and perform ANOVA using the function **aov** if Levene's test was not significant or Welch's ANOVA using the function **oneway.test** will be performed in case Levene's test was significant (both ANOVA functions are from the package **stats**). If the p-value of the ANOVA test is lower than or equal to the chosen significance level (α, 0.05 by default), repDilPCR will perform a post-hoc test: Tukey's all-pairs comparison test if Levene's test was not significant or an alternative test if Levene's test was significant. The alternative test will be Dunnett's T3 all-pairs comparison test for smaller sample sizes (<50 per group) or Games-Howell all-pairs comparison test for larger sample sizes. The post-hoc tests are respectively performed by the functions **tukeyTest**, **dunnettT3Test** and **gamesHowellTest** from the package **PMCMRplus**. Post-hoc tests automatically take care of the multiple testing problem, so reported p-values don't need adjustment when considered at the level of a single plot (single gene of interest). If you have evaluated many genes of interest, you might need additional correction for multiple testing depending on your research question.

When the options **all pairs** and **non-parametric** are selected, repDilPCR will first perform the Kruskal-Wallis rank sum test using the function **kruskalTest** from the package **PMCMRplus**. If the p-value of the Kruskal-Wallis test is lower than or equal to the chosen significance level (α, 0.05 by default), repDilPCR will perform Dunn's non-parametric all-pairs comparison test as a post-hoc test using the function **kwAllPairsDunnTest** from the package **PMCMRplus**. Post-hoc tests automatically take care of the multiple testing problem, so reported p-values don't need adjustment when considered at the level of a single plot (single gene of interest). If you have evaluated many genes of interest, you might need additional correction for multiple testing depending on your research question.

When the options **selected pairs** and **parametric** are selected, repDilPCR will first evaluate whether variance among experimental groups is homogenous by using Levene's test (the function **leveneTest** from the package **car**) and then perform unpaired two-sided pairwise t-tests using the function **pairwise.t.test** from the package **stats**). The pairwise t-tests will be performed with pooled variance in case Levene's test was not significant. Reported p-values from the pairwise t-tests will be adjusted for multiple testing using the Bonferroni correction at the level of each gene of interest (each single plot). If you have evaluated many genes of interest, you might need additional correction for multiple testing depending on your research question.

When the options **`selected pairs`** and **`non-parametric`** are selected, repDilPCR will conduct unpaired pairwise Wilcoxon rank sum tests using the function **`pairwise.wilcox.test`** from the package **`stats`**. Reported p-values from the pairwise Wilcoxon rank sum tests will be adjusted for multiple testing using the Bonferroni correction at the level of each gene of interest (each single plot). If you have evaluated many genes of interest, you might need additional correction for multiple testing depending on your research question.

The functions **`dunnettTest`**, **`tamhaneDunnettTest`**, **`kwManyOneDunnTest`** and **`tukeyTest`** depend on a random number generator to perform their calculations, which can lead to slightly different results when the same data is analyzed multiple times. In order to make repDilPCR return reproducible results when the same dataset is analyzed multiple times, the random number generator state is kept constant by using a hard-coded seed **`set.seed(40075017)`**.

5 Validation

We analyzed two qPCR experiments using three different methods in parallel: the dilution-replicate approach with repDilPCR, the classical standard curve approach (Pfaffl, 2001) and LinRegPCR (Ruijter, et al., 2009). The comparison of the three analysis methods showed that the obtained results were very similar when mRNA was used as a template (Validation experiment 1). In the other experiment, the aim was to assess expression levels of miRNAs (Validation experiment 2). LinRegPCR did not yield satisfactory results in this case, whereas repDilPCR performed as well as the standard curve method. In a third experiment, we verified the reproducibility of the dilution-replicate method by analysing the same samples in three separate PCR runs (Validation experiment 3). Additionally, we demonstrated successful inter-run normalization using different multi-plate experiment designs – sample maximization or target maximization.

5.1 Validation Experiment 1 (comparison with other methods)

5.1.1 Materials and methods

5.1.1.1 Cell lines

Three cell lines were used: HG-3, derived from a patient with chronic lymphocytic leukaemia (CLL); HG-3 D5, created in our lab from the parental HG-3 cell line by knocking out the *VHL* gene by CRISPR-Cas9; and LCL-Wei (B lymphoblastoid cell line derived from normal B cells). The HG-3 and LCL-Wei cell lines were obtained from DSMZ, Braunschweig, Germany, and were cultured according to the recommendations. Culture medium, fetal bovine serum and L-glutamine were acquired from Biochrom, Berlin, Germany. Each cell line was cultured in biological triplicates.

5.1.1.2 Reverse transcription and quantitative polymerase chain reaction (RT-qPCR)

Total RNA was independently isolated from all replicates using the RNeasy Mini Kit (Qiagen, Hilden, Germany), including treatment with DNase. Reverse transcription of RNA to cDNA was carried out using random primers and the SuperScript II reverse transcriptase (Life Technologies). An aliquot of each cDNA preparation was diluted twofold with water (the diluted samples were meant to be used as samples containing half the number of transcripts of the genes of interest per unit of volume, thus giving the opportunity to check whether the estimations by the different methods would deviate from the expected value: 50% of that in the undiluted sample). Each cDNA sample (original and twofold diluted) was additionally subjected to 5-fold serial dilution in two steps to prepare the dilution replicates needed by the dilution-replicate approach. In the end, of each biological replicate there were 1-fold, 2-fold, 5-fold, 10-fold, 25-fold and 50-fold diluted cDNA preparations. Additionally, a mix of cDNA samples was subjected to 5-fold serial dilution in 5 steps to prepare 6-point standard curves for the conventional

standard curve method. Reactions (10 µl) were set up in a 384-well plate in technical triplicates with aliquots (2 µl) of all of the prepared dilutions. The primers were synthesized by Eurofins Genomics, Ebersberg, Germany (Table S1). *TBP* and *LMNB1* were used as reference genes. *VHL* (von Hippel-Lindau tumour suppressor) and *LGALS1* (galectin 1) were the genes of interest. The SYBR Green Master Mix (Bio-Rad) was used to complete the reaction mixes. The plate was run on a QuantStudio 5 Real-Time PCR system (ThermoFisher Scientific). An initial polymerase activation step (15 min at 95 °C) was followed by 40 cycles consisting of 15 s at 95 °C and 1 min at 60 °C.

Table S1. Primer sequences (5’->3’)

Gene symbol	Forward primer	Reverse primer
Genes of interest		
VHL	CGTATGGCTCAACTTCGACG	TAACCAGAAGCCCATCGTGT
LGALS1	CGAGTGCGAGGCGAGGTG	CGTTGAAGCGAGGGTTGAAGTG
Reference genes		
LMNB1	CTGGAAATGTTTGCATCGAAGA	GCCTCCCATTGGTTGATCC
TBP	CCCGAAACGCCGAATATAA	GAAAATCAGTGCCGTGGTTC

5.1.1.3 Data analysis

The QuantStudio Design & Analysis software v. 1.5.1 (ThermoFisher Scientific) was used for initial quality control and to export data for downstream processing according to the requirements of each of the three analysis methods.

- For the standard curve method: QuantStudio Design & Analysis was used for automatic background subtraction and fitting 6-point standard curves using the data from the respective wells on the plate. The standard curves served to calculate the efficiency of the reaction for each primer pair, which was then used to calculate relative quantities of the template in each well of the validation experiment. Mean relative quantities at the level of technical replicates (only from wells with undiluted and 2-fold diluted cDNA) were exported and processed further using Microsoft Excel. First, normalization factors were calculated for each biological replicate by geometric averaging of the relative expression values of the two reference genes. For the undiluted wells, relative expression values of the genes of interest were normalized by dividing by the respective normalization factor. For the 2-fold diluted wells, relative expression values of the genes of interest were normalized by dividing by the normalization factors obtained from the respective undiluted wells. (Dilution reduces the concentration of all cDNA species, no matter whether they originate from genes of interest or from reference genes, consequently proper intra-well normalization would yield the same result for expression of the gene of interest in a diluted and in an undiluted sample. Normalization of expression values of genes of interest in a 2-fold diluted sample to reference genes in the respective undiluted sample should result in relative quantities that are approximately 50% of those determined in the undiluted sample. In our case, this is what we want and expect and getting such a result would mean that the analytical method is capable of accurate quantitative assessment.) To make comparisons more convenient, normalized expression values were converted to $\log_2$ scale, averaged across biological replicates and plotted on a graph, taking the undiluted wild-type HG-3 cells sample (HG-3 WT) as a reference (expression level of 1 on a linear scale or 0 on a logarithmic scale).

- For the LinRegPCR method: fluorescence data per cycle without baseline correction (Rn values) were exported from QuantStudio Design & Analysis and imported into LinRegPCR (only from wells with undiluted and 2-fold diluted cDNA). LinRegPCR was used for baseline estimation and for determination of windows of linearity, efficiencies of individual reactions and mean efficiency per amplicon group. The latter was used by the program to calculate the N_0 values (relative quantities). They were exported to Microsoft Excel, averaged at the level of technical replicates and processed further similarly to relative quantities obtained by the standard curve method. First, normalization factors were calculated for each biological replicate by geometric averaging of the relative expression values of the two reference genes. For the undiluted wells, relative expression values of the genes of interest were normalized by dividing by the respective normalization factor. For the 2-fold diluted wells, relative expression values of the genes of interest were normalized by dividing by the normalization factors obtained from the respective undiluted wells. (Dilution reduces the concentration of all cDNA species, no matter whether they originate from genes of interest or from reference genes, consequently proper intra-well normalization would yield the same result for expression of the gene of interest in a diluted and in an undiluted sample. Normalization of expression values of genes of interest in a 2-fold diluted sample to reference genes in the respective undiluted sample should result in relative quantities that are approximately 50% of those determined in the undiluted sample. In our case, this is what we want and expect and getting such a result would mean that the analytical method is capable of accurate quantitative assessment.) To make comparisons more convenient, normalized expression values were converted to $\log_2$ scale, averaged across biological replicates and plotted on a graph, taking the undiluted wild-type HG-3 cells sample (HG-3 WT) as a reference (expression level of 1 on a linear scale or 0 on a logarithmic scale).
- For the dilution-replicate method with repDilPCR: QuantStudio Design & Analysis was used for automatic background subtraction. Then, a common threshold was chosen for all amplicons (0.3) and the Cq values were exported and arranged in a CSV file according to the format required by repDilPCR. Similarly to the procedures above, Cq values of reference genes from wells with 2-fold, 10-fold and 50-fold dilution were replaced by the Cq values from the respective undiluted, 5-fold diluted or 25-fold diluted wells. The CSV file was imported into repDilPCR and automatic analysis was performed using two reference genes while all other settings were kept at their default values. Normalized expression values were plotted on a $\log_2$ scale and the graph was exported in the PNG file format.

Mean expression values obtained by the three analysis methods were used to construct scatter plots with regression lines for each pair of methods per gene. The parameters of the regression (slope, R^2, p-value) were used to estimate the agreement between the different methods.

5.1.2 Results

The analysis of the experiment using the three different approaches (standard curve, LinRegPCR and repDilPCR) showed that all of them yielded similar results for both of the investigated genes of interest: *VHL* (Fig. S9) and *LGALS1* (Fig. S10, identical with Fig. 2 from the main text). The difference in gene expression between the undiluted and the twofold diluted sample of each cell line would be expected to be 1 unit on a $\log_2$ scale. The figures show that these differences are indeed close to 1 for all of the three analysis approaches. As expected, the HG-3 D5 cell line had significantly lower expression of *VHL* than the parental HG-3 WT cell line (Fig. S9).

Pairwise scatter plots with regression lines show the goodness of the correlation between the methods quantitatively (Fig. S11, identical with Fig. 3 from the main text). The coefficient of determination was high for each pairwise comparison but results from repDilPCR correlated a little bit better with results obtained via the standard curve method (R^2 = 0.95-0.96) than with results from LinRegPCR (R^2 = 0.91-0.95).

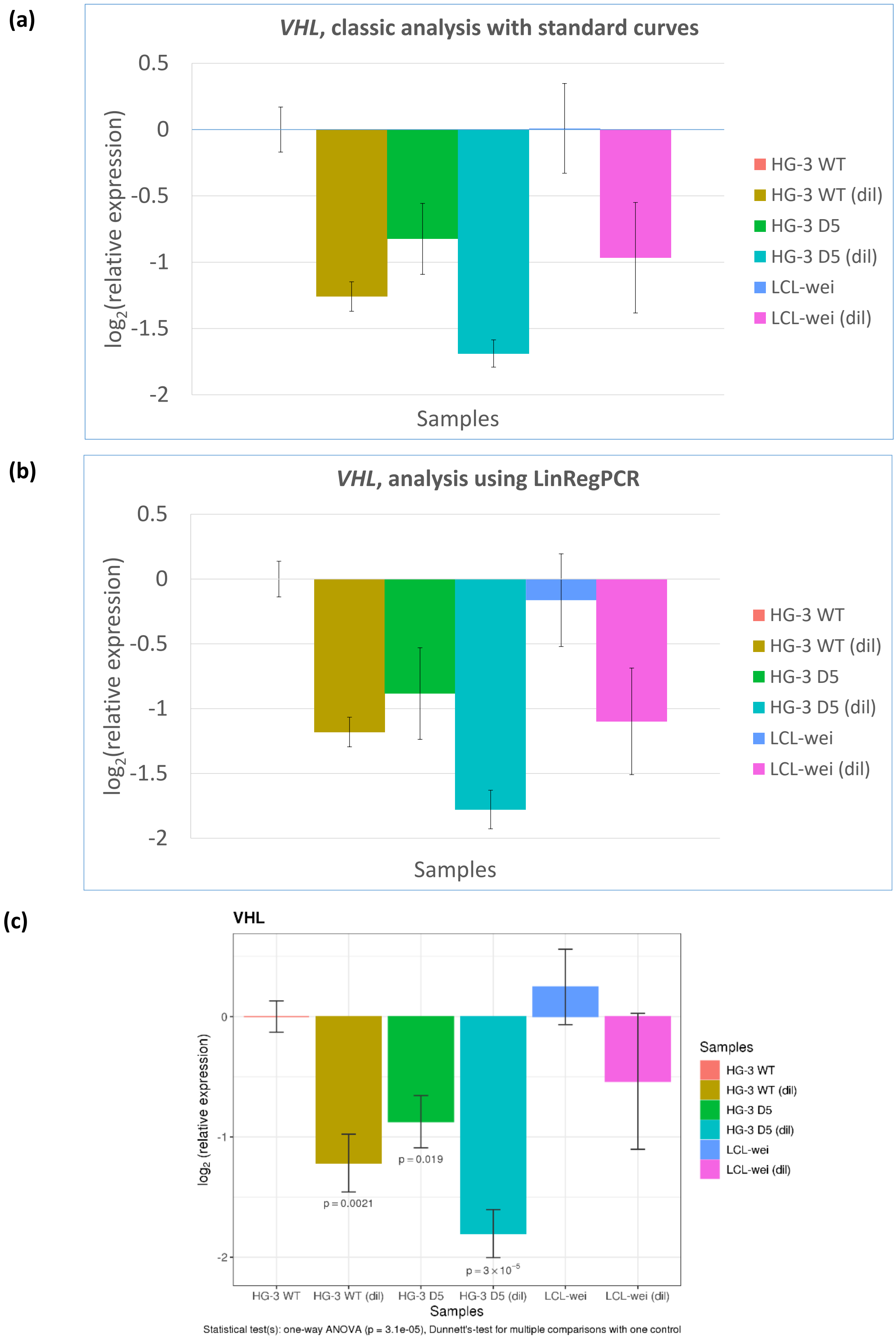

Figure S9: Relative expression of *VHL* in different cell lines in Validation Experiment 1 as determined according to three different approaches: (a) standard curve; (b) LinRegPCR and (c) repDilPCR. Graphs **(a)** and **(b)** were prepared manually using Microsoft Excel. Graph **(c)** was prepared fully automatically by repDilPCR using unprocessed Cq values. P-values are for comparisons with HG-3 WT. The twofold diluted samples are denoted by ”(dil)” after the name of the respective cell line.

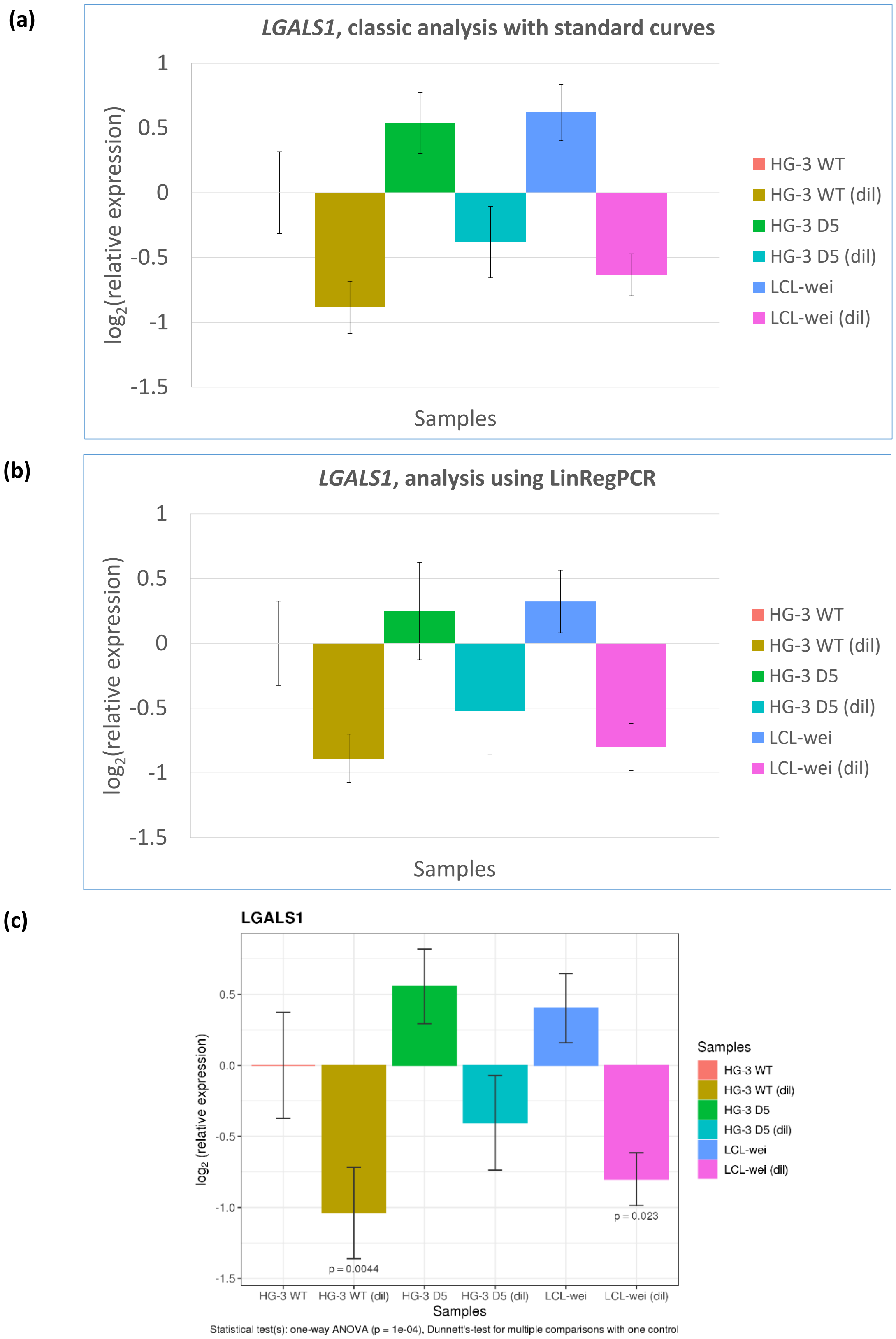

Figure S10: Relative expression of *LGALS1* in different cell lines in Validation Experiment 1 as determined according to three different approaches: (a) standard curve; (b) LinRegPCR and (c) repDilPCR. Graphs **(a)** and **(b)** were prepared manually using Microsoft Excel. Graph **(c)** was prepared fully automatically by repDilPCR using unprocessed Cq values. P-values are for comparisons with HG-3 WT. The twofold diluted samples are denoted by ”(dil)” after the name of the respective cell line.

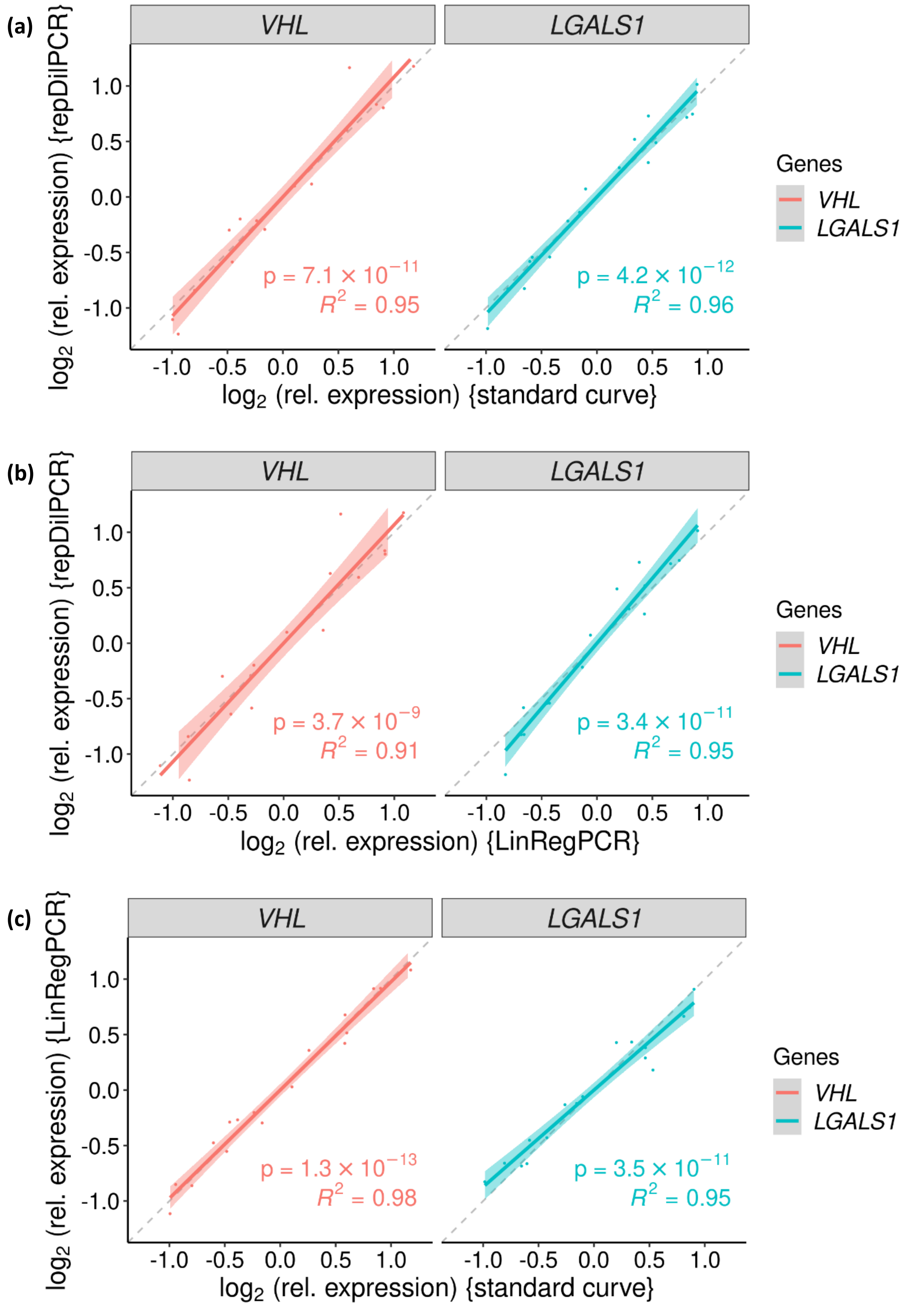

Figure S11: Pairwise scatter plots comparing relative gene expression (on $\log_2$ scale) determined by three different approaches in Validation Experiment 1. (a) Comparison of repDilPCR against the standard curve method. (b) Comparison of repDilPCR against LinRegPCR. (c) Comparison of LinRegPCR against the standard curve method. Each comparison was performed separately for the two genes of interest that were analyzed: *VHL* and *LGALS1*. Each dot corresponds to a biological replicate in the experiment. The data were scaled to set the mean $\log_2$(relative expression) to 0 to make comparisons possible. Linear regression was performed and the regression line with its 95%-confidence interval is shown for each comparison, as well as the p-value and the coefficient of determination (R^2) of the model. Dashed lines indicate perfect correlation.

5.2 Validation Experiment 2 (comparison with other methods)

5.2.1 Materials and methods

5.2.1.1 Cell lines

Three cell lines were used: MEC-155 and HG-3, originating from CLL, and Jurkat, derived from a patient with T cell leukaemia. The HG-3 and Jurkat cell lines were obtained from DSMZ, Braunschweig, Germany, and were cultured according to the recommendations (90% RPMI-1640 medium + 10% FBS). MEC-155 cells were a kind gift from Martina Seiffert (German Cancer Research Center, Heidelberg) and were derived from the parental MEC-1 cell line by transfecting with an expression vector for miR-155. They were cultured in Iscove's MDM medium supplemented with FBS (10%). Culture medium, FBS and L-glutamine were acquired from Biochrom, Berlin, Germany. Each cell line was cultured in biological triplicates.

5.2.1.2 Reverse transcription and quantitative polymerase chain reaction (RT-qPCR)

Total RNA was independently isolated from all replicates using the RNeasy Mini Kit (Qiagen, Hilden, Germany), including treatment with DNase. Reverse transcription to cDNA was carried out using the miScript II RT Kit with the miScript HiSpec buffer (Qiagen). An aliquot of each cDNA preparation was diluted twofold with water (the diluted samples were meant to be used as samples containing half the number of transcripts of the genes of interest per unit of volume, thus giving the opportunity to check whether the estimations by the different methods would deviate from the expected value: 50% of that in the undiluted sample). Each cDNA sample (original and twofold diluted) was additionally subjected to 5-fold serial dilution in two steps to prepare the dilution replicates needed by the dilution-replicate approach. In the end, of each biological replicate there were 1-fold, 2-fold, 5-fold, 10-fold, 25-fold and 50-fold diluted cDNA preparations. Additionally, a mix of cDNA samples was subjected to 5-fold serial dilution in 5 steps to prepare 6-point standard curves for the conventional standard curve method. Reactions (10 µl) were set up in a 384-well plate in technical triplicates with aliquots (2 µl) of all of the prepared dilutions. *SNORD95, SNORD68* and *RNU6-2* were used as reference genes. *MIR17* (microRNA 17) and *MIR155* (microRNA 155) were the genes of interest. Forward primers were ordered from Qiagen as miScript Primer Assays (catalog numbers MS00033726, MS00033712, MS00033740 and MS00029274) with the exception of the forward primer for miR-155 which was synthesized by Eurofins Genomics, Ebersberg, Germany (sequence: 5`-TTAATGCTAATCGTGATAGGGGTAA-3`). The QuantiTect SYBR Green PCR Master Mix and the miScript Universal Primer (Qiagen) were used to complete the reaction mixes. The plate was run on a QuantStudio 5 Real-Time PCR system (ThermoFisher Scientific). An initial polymerase activation step (15 min at 95 °C) was followed by 40 cycles consisting of 15 s at 95 °C, 30 s at 53 °C and 30 s at 70 °C.

5.2.1.3 Data analysis

The QuantStudio Design & Analysis software v. 1.5.1 (ThermoFisher Scientific) was used for initial quality control and to export data for downstream processing according to the requirements of each of the three analysis methods.

- For the standard curve method: QuantStudio Design & Analysis was used for automatic background subtraction and fitting 6-point standard curves using the data from the respective wells on the plate. The standard curves served to calculate the efficiency of the reaction for each primer pair, which was then used to calculate relative quantities of the template in each well of the validation experiment. Mean relative quantities at the level of technical replicates (only from wells with undiluted and 2-fold diluted cDNA) were exported and processed further using Microsoft Excel. First, normalization factors were calculated for each biological replicate by geometric averaging of the relative expression values of the three reference genes. For the undiluted wells, relative expression values of the genes of interest were normalized by dividing by the respective normalization factor. For the 2-fold diluted wells, relative expression values of the genes of interest were normalized by dividing by the normalization factors obtained from

the respective undiluted wells. (Dilution reduces the concentration of all cDNA species, no matter whether they originate from genes of interest or from reference genes, consequently proper intra-well normalization would yield the same result for expression of the gene of interest in a diluted and in an undiluted sample. Normalization of expression values of genes of interest in a 2-fold diluted sample to reference genes in the respective undiluted sample should result in relative quantities that are approximately 50% of those determined in the undiluted sample. In our case, this is what we want and expect and getting such a result would mean that the analytical method is capable of accurate quantitative assessment.) To make comparisons more convenient, normalized expression values were converted to log_2 scale, averaged across biological replicates and plotted on a graph, taking the undiluted MEC-155 cells sample as a reference (expression level of 1 on a linear scale or 0 on a logarithmic scale).

- For the LinRegPCR method: fluorescence data per cycle without baseline correction (Rn values) were exported from QuantStudio Design & Analysis and imported into LinRegPCR (only from wells with undiluted and 2-fold diluted cDNA). LinRegPCR was used for baseline estimation and for determination of windows of linearity, efficiencies of individual reactions and mean efficiency per amplicon group. The latter was used by the program to calculate the N_0 values (relative quantities). They were exported to Microsoft Excel, averaged at the level of technical replicates and processed further similarly to relative quantities obtained by the standard curve method. First, normalization factors were calculated for each biological replicate by geometric averaging of the relative expression values of the three reference genes. For the undiluted wells, relative expression values of the genes of interest were normalized by dividing by the respective normalization factor. For the 2-fold diluted wells, relative expression values of the genes of interest were normalized by dividing by the normalization factors obtained from the respective undiluted wells. (Dilution reduces the concentration of all cDNA species, no matter whether they originate from genes of interest or from reference genes, consequently proper intra-well normalization would yield the same result for expression of the gene of interest in a diluted and in an undiluted sample. Normalization of expression values of genes of interest in a 2-fold diluted sample to reference genes in the respective undiluted sample should result in relative quantities that are approximately 50% of those determined in the undiluted sample. In our case, this is what we want and expect and getting such a result would mean that the analytical method is capable of accurate quantitative assessment.) To make comparisons more convenient, normalized expression values were converted to log_2 scale, averaged across biological replicates and plotted on a graph, taking the undiluted MEC-155 cells sample as a reference (expression level of 1 on a linear scale or 0 on a logarithmic scale).
- For the dilution-replicate method with repDilPCR: QuantStudio Design & Analysis was used for automatic background subtraction. Then, a common threshold was chosen for all amplicons (0.06) and the Cq values were exported and arranged in a CSV file according to the format required by repDilPCR. Similarly to the procedures above, Cq values of reference genes from wells with 2-fold, 10-fold and 50-fold dilution were replaced by the Cq values from the respective undiluted, 5-fold diluted or 25-fold diluted wells. The CSV file was imported into repDilPCR and automatic analysis was performed with the default settings. Normalized expression values were plotted on a log_2 scale and the graph was exported in the PNG file format.

Mean expression values obtained by the three analysis methods were used to construct scatter plots with regression lines for each pair of methods per gene. The parameters of the regression (slope, R^2, p-value) were used to estimate the agreement between the different methods.

5.2.2 Results

The analysis of the experiment using the three different approaches (standard curve, LinRegPCR and repDilPCR) showed that all of them yielded similar results for miR-17 (Fig. S12).

(a)

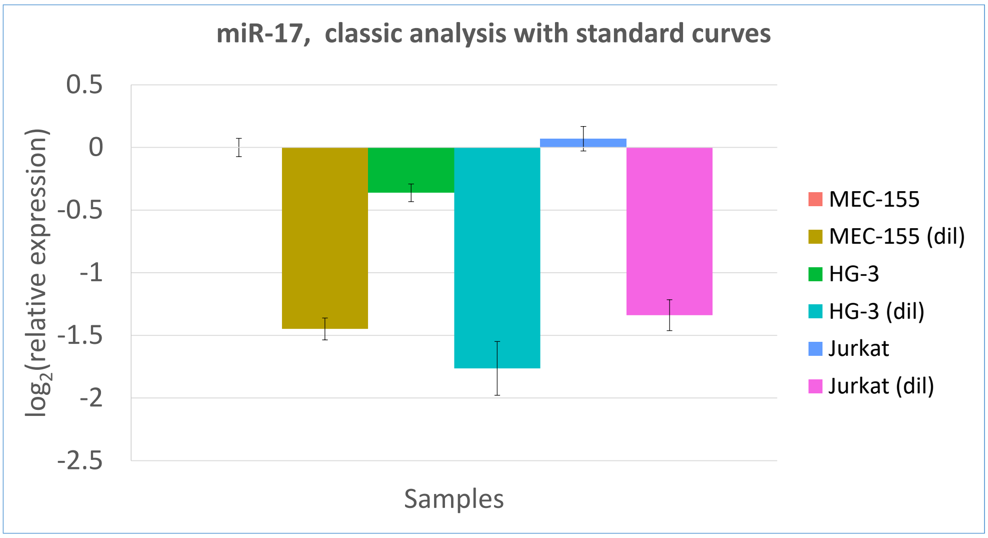

(b)

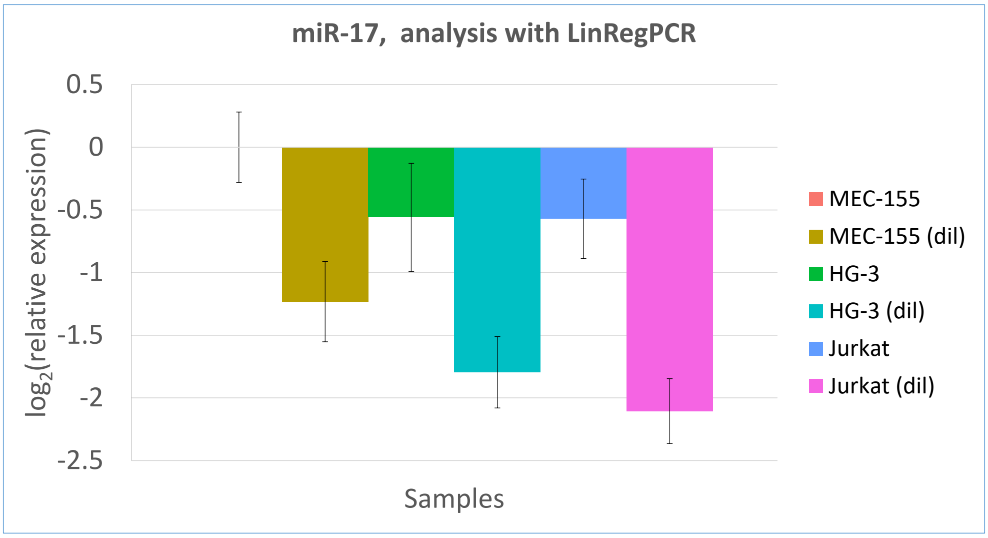

(c)

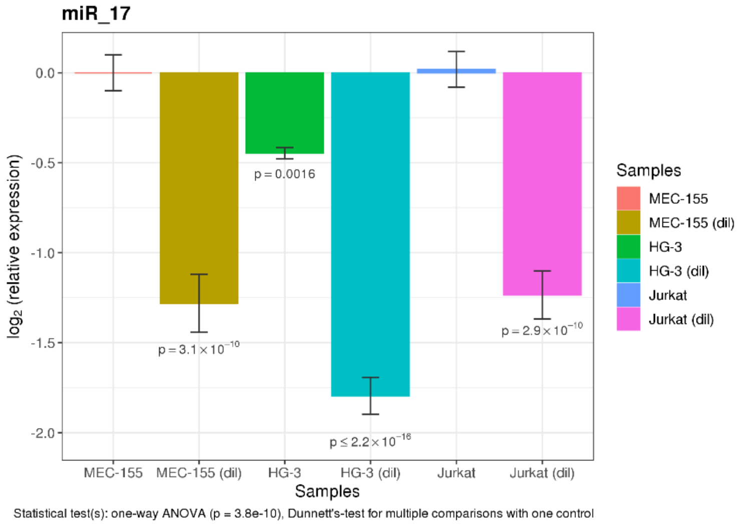

Figure S12: Relative expression of miR-17 in different cell lines in Validation Experiment 2 as determined according to three different approaches: (a) standard curve; (b) LinRegPCR and (c) repDilPCR. Graphs **(a)** and **(b)** were prepared manually using Microsoft Excel. Graph **(c)** was prepared fully automatically by repDilPCR using unprocessed Cq values. P-values are for comparisons with MEC-155. The twofold diluted samples are denoted by ”(dil)” after the name of the respective cell line.

(a)

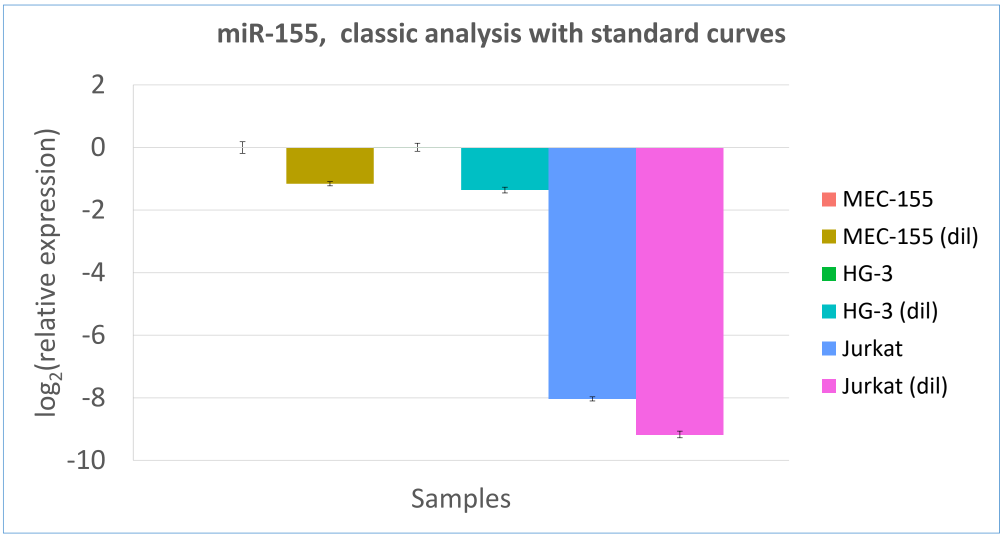

(b)

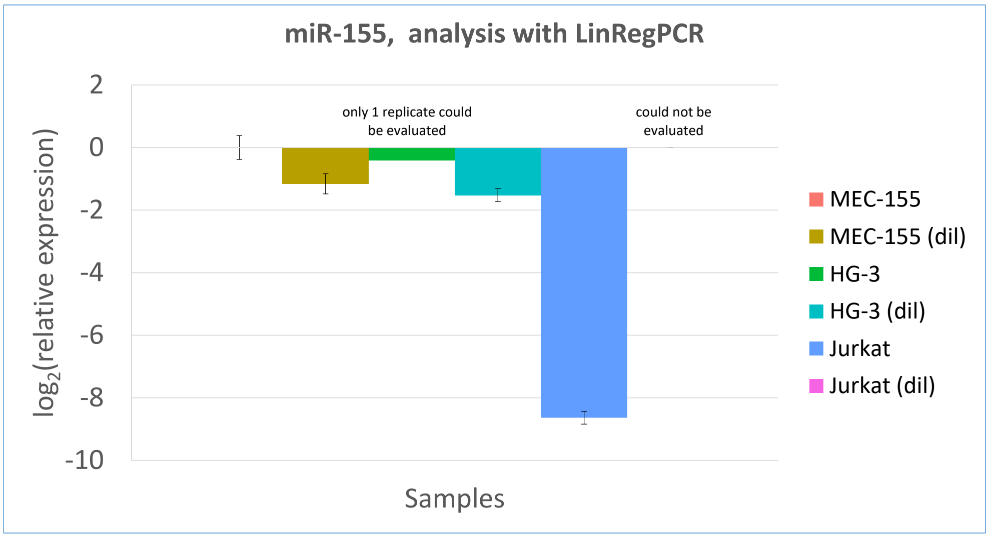

(c)

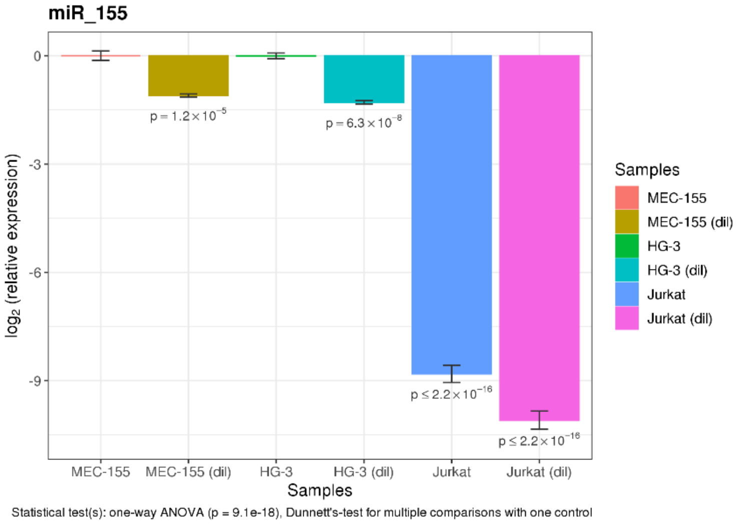

Figure S13: Relative expression of miR-155 in different cell lines in Validation Experiment 2 as determined according to three different approaches: (a) standard curve; (b) LinRegPCR and (c) repDilPCR. Graphs **(a)** and **(b)** were prepared manually using Microsoft Excel. Graph **(c)** was prepared fully automatically by repDilPCR using unprocessed Cq values. P-values are for comparisons with MEC-155. The twofold diluted samples are denoted by ”(dil)” after the name of the respective cell line.

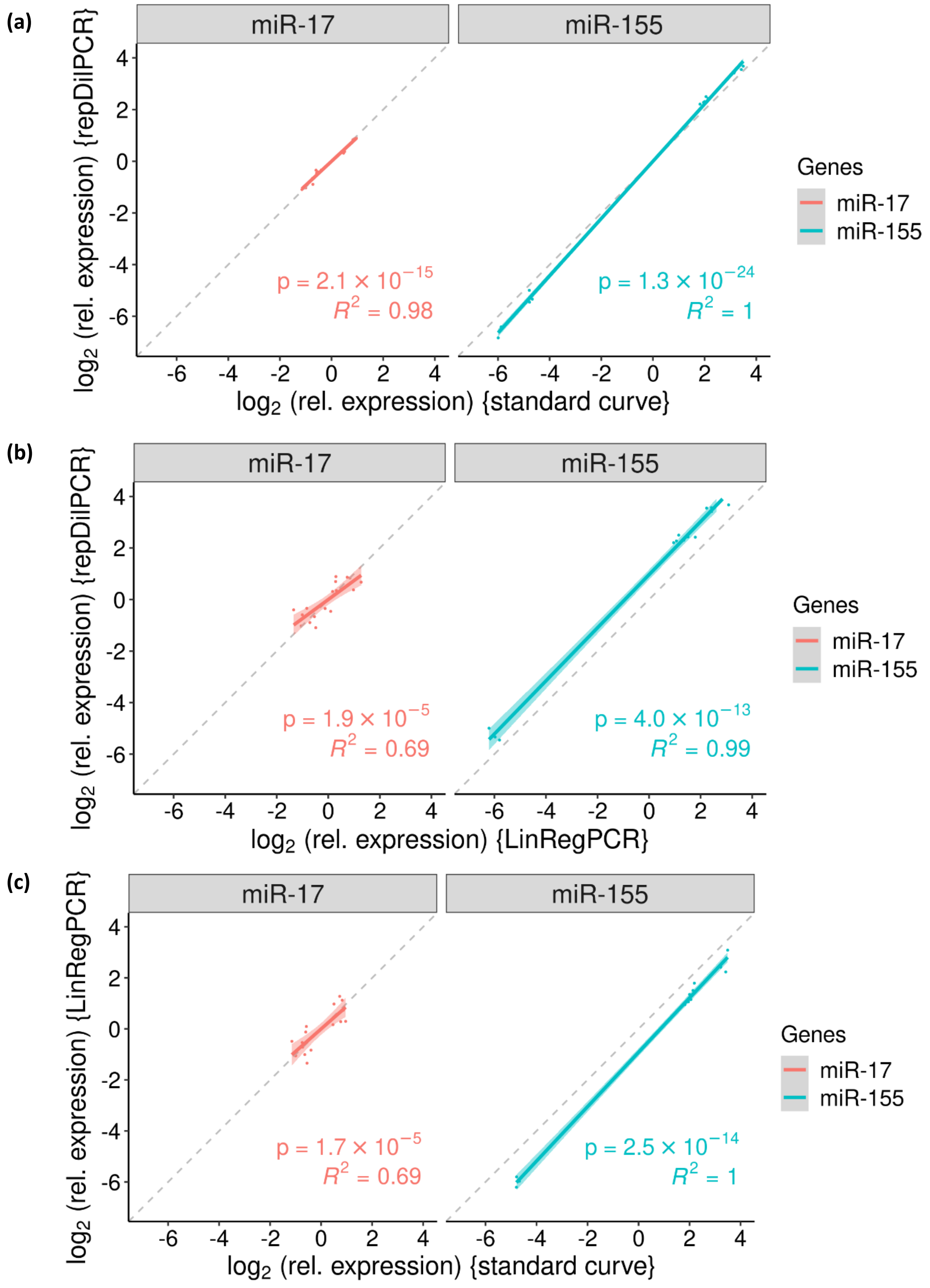

Figure S14: Pairwise scatter plots comparing relative gene expression (on $\log_2$ scale) determined by three different approaches in Validation Experiment 2. (a) Comparison of repDilPCR against the standard curve method. (b) Comparison of repDilPCR against LinRegPCR. (c) Comparison of LinRegPCR against the standard curve method. Each comparison was performed separately for the two miRNAs that were analyzed: miR-17 and miR-155. Each dot corresponds to a biological replicate in the experiment. The data were scaled to set the mean $\log_2$(relative expression) to 0 to make comparisons possible. Linear regression was performed and the regression line with its 95%-confidence interval is shown for each comparison, as well as the p-value and the coefficient of determination (R^2) of the model. Dashed lines indicate perfect correlation.

However, results obtained by LinRegPCR had visibly larger standard deviations. We attribute this to our observation that amplification curves resulting from miRNA templates with the usage of the miScript PCR system looked flatter and had shorter linear parts than amplification curves for normal mRNA assays, which made it difficult for the LinRegPCR program to determine windows of linearity reliably. This problem was especially pronounced with miR-155, in which case 5 of the amplification curves could not be evaluated at all (2 replicates of the HG-3 sample and all 3 replicates of the Jurkat (dil) sample; Fig. S13b). The standard curve and the dilution-replicate method (repDilPCR) were not affected by the flatter amplification curves and yielded similar results for miR-155, too (Fig. S13a and S13c). The difference in gene expression between the undiluted and the twofold diluted sample of each cell line would be expected to be 1 unit on a $\log_2$ scale. The figures show that these differences are somewhat larger than 1 in this experiment as opposed to Validation Experiment 1. A possible explanation could be higher loss of template during preparation of dilutions in Validation Experiment 2, as a higher number of shorter DNA molecules could stick to the surface of pipette tips and thus be excluded from the subsequent reaction.

Pairwise scatter plots with regression lines show the goodness of the correlation between the methods quantitatively (Fig. S14, identical with Fig. 4 from the main text). Results from repDilPCR and the standard curve method correlated very well together (R^2 = 0.98-1.0), whereas correlation was weaker for comparisons of LinRegPCR with the other two methods (R^2 was 0.69 for miR-17 and 0.99-1.0 for miR-155 but the regression lines for miR-155 were shifted away from the diagonal because several samples could not be evaluated with LinRegPCR and the missing data interfered with the centering of the dataset around 0).

5.3 Validation Experiment 3 (reproducibility and inter-run calibration)

5.3.1 Materials and methods

5.3.1.1 Cells

Three cell lines were used: HeLa, derived from a patient with cervix adenocarcinoma; HEK-293, established from a human primary embryonal kidney; and HS-5 (transformed bone marow fibroblasts). The HeLa and HEK-293 cell lines were obtained from DSMZ, Braunschweig, Germany, whereas HS-5 was purchased from ATCC. All cell lines were cultured according to the respective recommendations. Culture medium, fetal bovine serum and L-glutamine were acquired from ThermoFisher Scientific. Each cell line was cultured in biological triplicates.

Blood was obtained from three CLL patients after informed consent in accordance with the Declaration of Helsinki and under a protocol approved by the ethical committee of the University of Ulm. Blood samples were subjected to density gradient centrifugation (Ficoll-Paque, Biochrom, Berlin, Germany) to isolate peripheral blood mononuclear cells (PBMCs).

5.3.1.2 Reverse transcription and quantitative polymerase chain reaction (RT-qPCR)

Total RNA was isolated from all cell line and patient samples using the RNeasy Mini Kit (Qiagen, Hilden, Germany), including treatment with DNase. Reverse transcription of RNA to cDNA was carried out using random primers and the SuperScript II reverse transcriptase (Life Technologies). Each cDNA sample was subjected to 5-fold serial dilution in two steps to prepare the dilution replicates needed by the dilution-replicate approach. In the end, there were 1-fold, 5-fold and 25-fold diluted cDNA preparations of each biological replicate. Reactions (10 µl) were set up in three identical 384-well plates with aliquots (2 µl) of all of the prepared dilutions. *HPRT* and *PPIA* were used as reference genes. *COL3A1* (alpha 1 chain of type III collagen), CXCR4 (fusin, CD184) and *LDHA* (lactate dehydrogenase A) were the genes of interest. The primers were synthesized by Eurofins Genomics, Ebersberg, Germany (Table S2), with the exception of the *LDHA* primers, which were purchased from Qiagen (QuantiTect Primer Assay, catalog No. 249900). The SYBR Green Master

Mix (Bio-Rad) was used to complete the reaction mixes. The plates were run on a QuantStudio 5 Real-Time PCR system (ThermoFisher Scientific). An initial polymerase activation step (15 min at 95 °C) was followed by 40 cycles consisting of 15 s at 95 °C and 1 min at 60 °C.

Table S2. Primer sequences (5’->3’)

Gene symbol	Forward primer	Reverse primer
Genes of interest		
COL3A1	CGCTCTGCTTCATCCCACTA	ATCCGCATAGGACTGACCAA
CXCR4	CTCCAAGCTGTCACACTCCA	TCGATGCTGATCCCAATGTA
Reference genes		
HPRT	TGTAGCCCTCTGTGTGCTCAAG	CCTGTTGACTGGTCATTACAATAGCT
PPIA	CGGGAGGCCAGGCTCGT	TGAAAGCAGGAACCCTTATAACCAA

5.3.1.3 Data analysis

The QuantStudio Design & Analysis software v. 1.5.1 (ThermoFisher Scientific) was used for initial quality control, automatic background subtraction, application of a common threshold for all amplicons (0.5) and export of data for downstream processing with repDilPCR. Normalized expression values were plotted on a $\log_2$ scale and the graphs were exported in the PNG file format.

5.3.2 Results and discussion

The three separate PCR runs yielded very similar results for each of the genes of interest: *COL3A1* (Fig. S15), *CXCR4* (Fig. S16) and *LDHA* (Fig. S17). This demonstrates the reproducibility of the dilution-replicate method.

Careful experimental planning is needed when the number of samples and target genes in an experiment is too high and the reactions cannot fit on a single PCR plate. Two different approaches are known – sample maximization and target (gene) maximization. Sample maximization means that as many samples as possible are analyzed in the same run, while with target maximization one strives to analyze a maximal number of targets in each run and samples are distributed across runs. In relative quantification studies, when the experimenter is interested in comparing the expression level of a particular gene between samples, it is usually recommended to follow the sample maximization method to eliminate the run-to-run variation between samples. Usage of the target maximization approach is also possible but more challenging statistically because it requires inter-run calibration. Different statistical methods have been published to perform such calibration. One of the most popular methods suggests the use of inter-run calibrators, samples that are analyzed in all runs and that serve to calculate correction factors to remove the run-to-run difference (Hellemans et al., 2007). This method states that inter-run calibration should be performed on a per gene basis. Another method makes use of all overlapping technical and biological replicates between runs and calculates a single correction factor per run (Ruijter et al., 2015).

As the dilution-replicate method depends on using the same threshold for different targets in an experiment, one should be cautious using the sample maximization approach because of the potential for instrument-related variation in detector sensitivity between runs. Nevertheless, we tested this approach using the data from the above experiment by normalizing GOIs from every run to either

reference genes from the same plate or to reference genes from any of the other two runs (Fig. S18, S19 and S20 for *COL3A1*, *CXCR4* and *LDHA*, respectively). Any observed bias when Cq values for

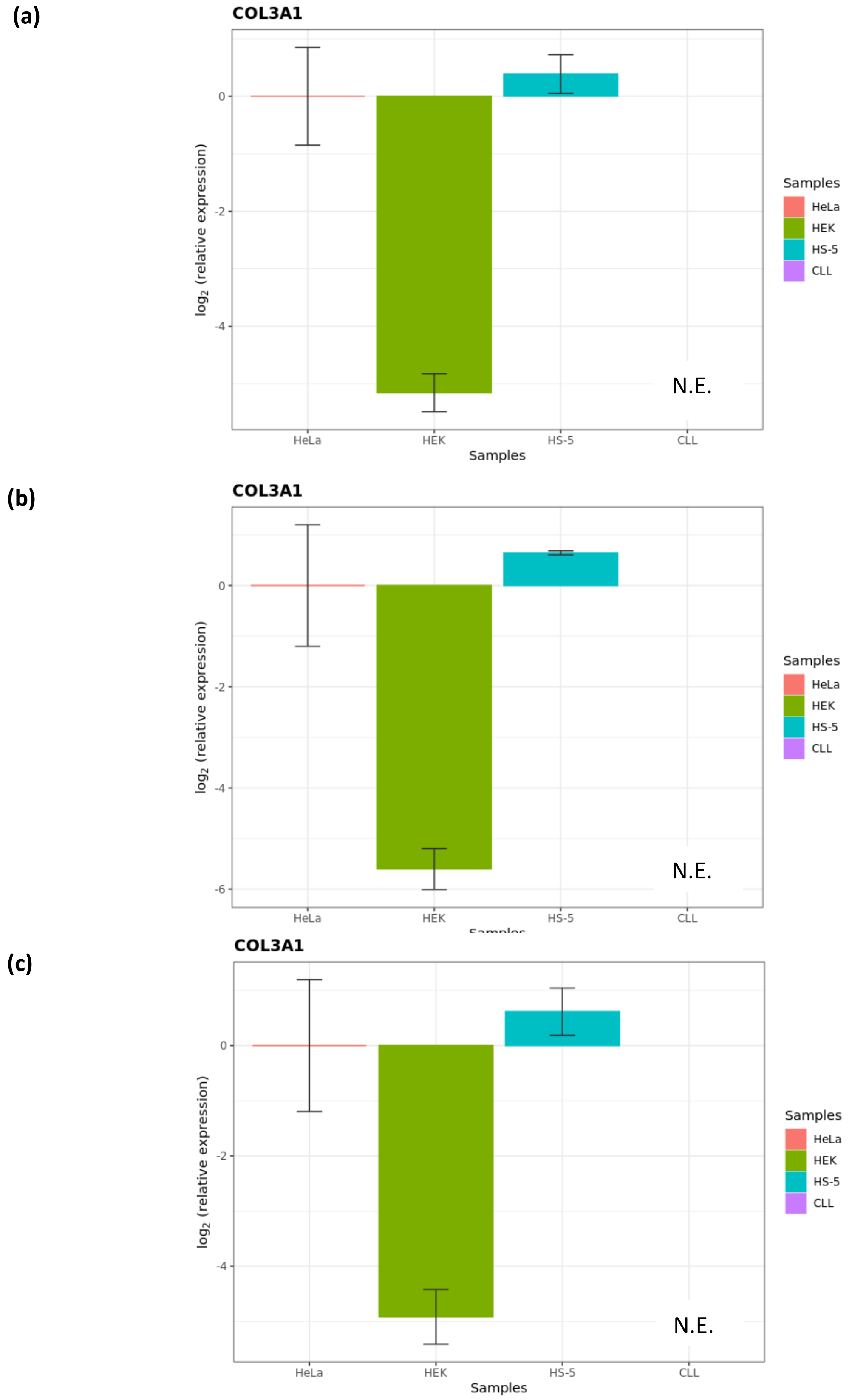

Figure S15: Relative expression of *COL3A1* in different cell lines and in CLL cells from patients. Results from three separate qPCR runs are shown: (a) run 1; (b) run 2; (c) run 3. Error bars depict standard deviation at the level of biological replicates (n=3). N.E., not expressed.

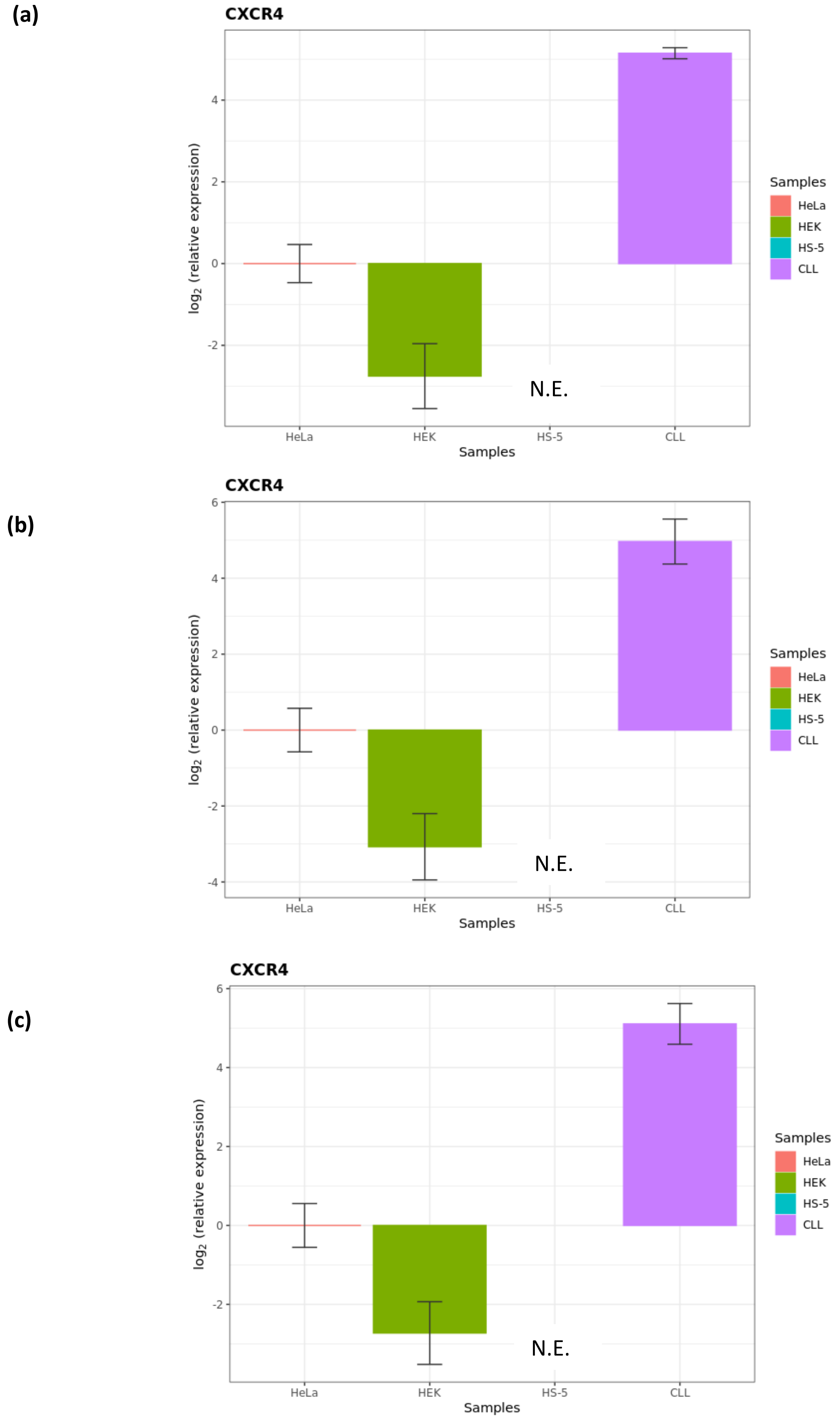

Figure S16: Relative expression of *CXCR4* in different cell lines and in CLL cells from patients. Results from three separate qPCR runs are shown: (a) run 1; (b) run 2; (c) run 3. Error bars depict standard deviation at the level of biological replicates (n=3). N.E., not expressed.

(a)

(b)

(c)

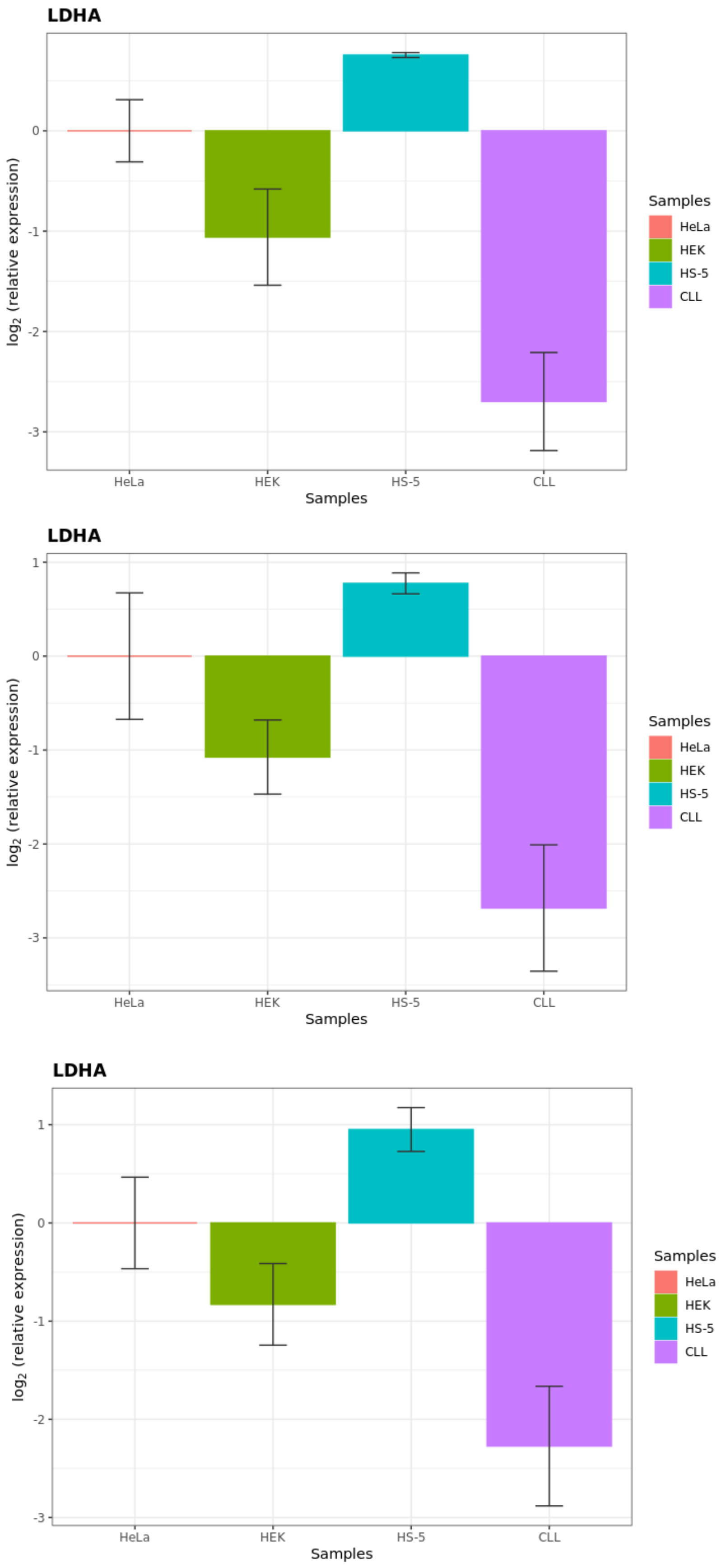

Figure S17: Relative expression of *LDHA* in different cell lines and in CLL cells from patients. Results from three separate qPCR runs are shown: (a) run 1; (b) run 2; (c) run 3. Error bars depict standard deviation at the level of biological replicates (n=3). N.E., not expressed.

reference genes from a different run were taken was quite small, indicating that the fluorescence detector sensitivity of our device was stable between runs (all performed on different days). Users should validate whether this is true also for their instrument if they wish to use the dilution-replicate method with the sample maximization strategy. If yes, the usage is simple: one just has to combine the Cq values from all runs (not forgetting the common threshold rule!) into a single CSV file and then process it with repDilPCR as a single dataset.

With large experiments planned according to the target maximization strategy, one should analyze each run with repDilPCR separately without normalizing to a reference sample (by removing the "default" text under the option **Reference sample or experimental group**) and then export the resulting relative expression values in tabular form. Thereafter, one can use Factor qPCR (Ruijter et al., 2015) to calculate correction factors (either on a per plate or on a per plate and target basis) and perform inter-run calibration. The corrected relative expression values can be fed back into repDilPCR for the final graphical presentation and statistical analysis. Here, we tested the two inter-run calibration methods mentioned above (Hellemans et al., 2007 and Ruijter et al., 2015). First of all, we plotted the results from the three separate qPCR runs on common plots without correction to demonstrate the inter-run variation (Fig. S21a, S22a and S23a for *COL3A1*, *CXCR4* and *LDHA*, respectively). The differences were most prominent for *CXCR4* (Fig. S22a). Correction on a per plate and target basis as suggested by Hellemans et al. functioned very well and neutralized the inter-run variation (Fig. S21b, S22b and S23b). In contrast, correction on a per plate basis did not give satisfactory results (Fig. S21c, S22c and S23c).

In summary, we recommend that multi-plate dilution-replicate qPCR experiments be planned according to the target maximization strategy and that subsequent inter-run calibration be performed on a per plate and target basis. The sample maximization strategy can also be used if it can be demonstrated that fluorescence measurements (resp. thresholds) are stable across runs.

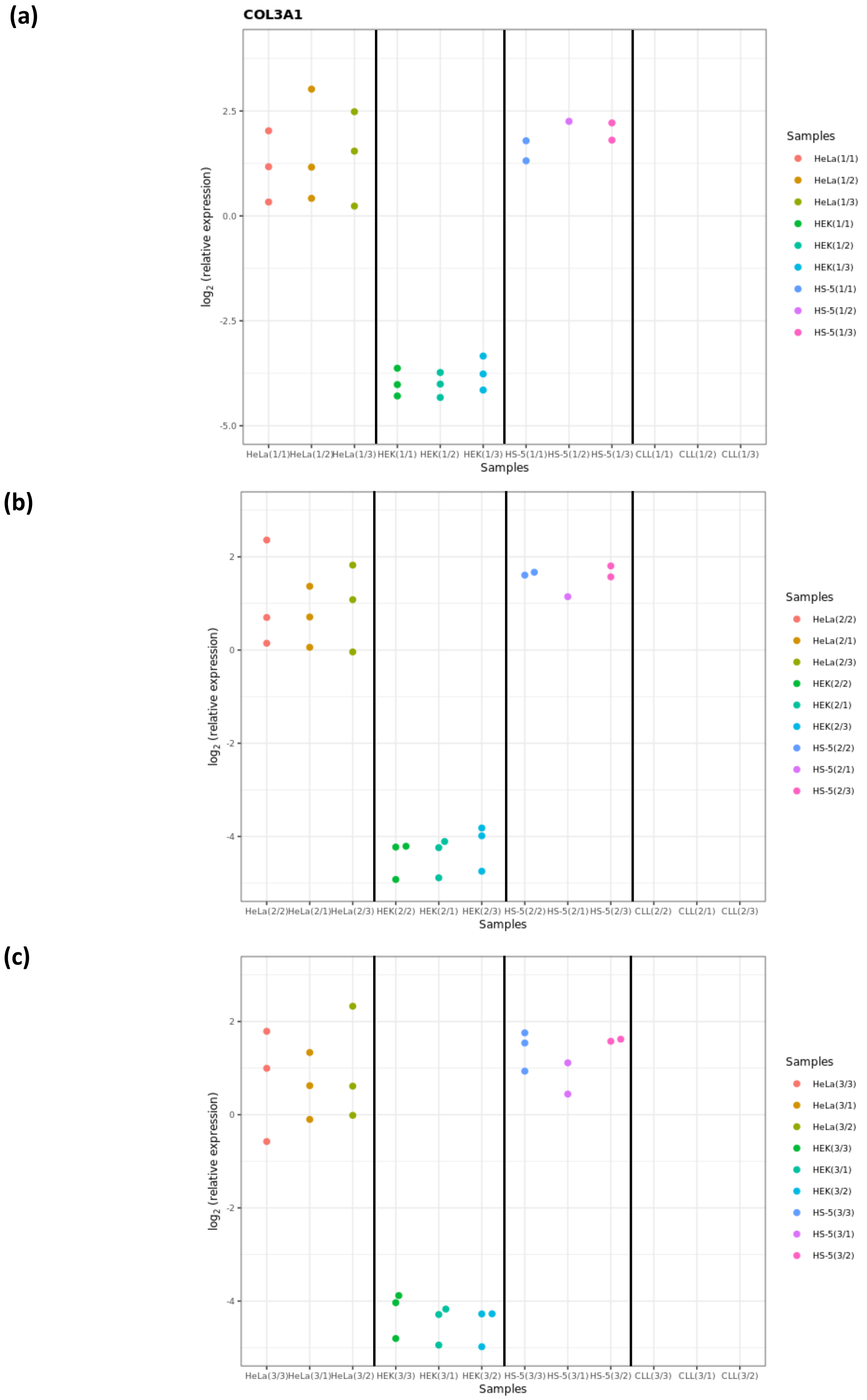

Figure S18: Comparison of relative expression of *COL3A1* in different cell lines and in CLL cells from patients when the reference genes (*HPRT* and *PPIA*) were measured either in the same run as *COL3A1* or in different runs: (a) *COL3A1* measured in run 1; (b) *COL3A1* measured in run 2; (c) *COL3A1* measured in run 3. Each of the panel subsections divided by vertical lines summarizes results for one cell type. The leftmost result in each subsection pertains to the case when *COL3A1* and the RGs were measured on the same plate; the middle and right results pertain to cases when RGs were measured on one of the other two plates. Each particular combination is denoted by numbers in brackets after the sample names, where the numerator is the number of the run in which *COL3A1* was measured and the denominator is the number of the run in which the RGs were measured.

(a)

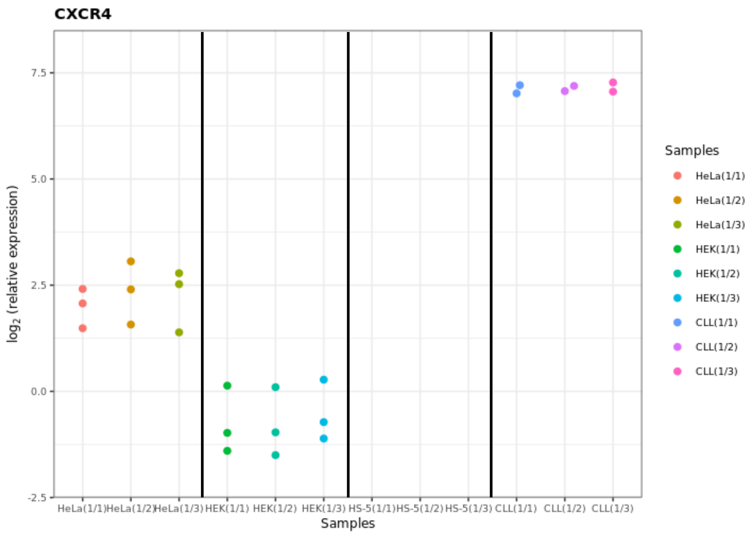

(b)

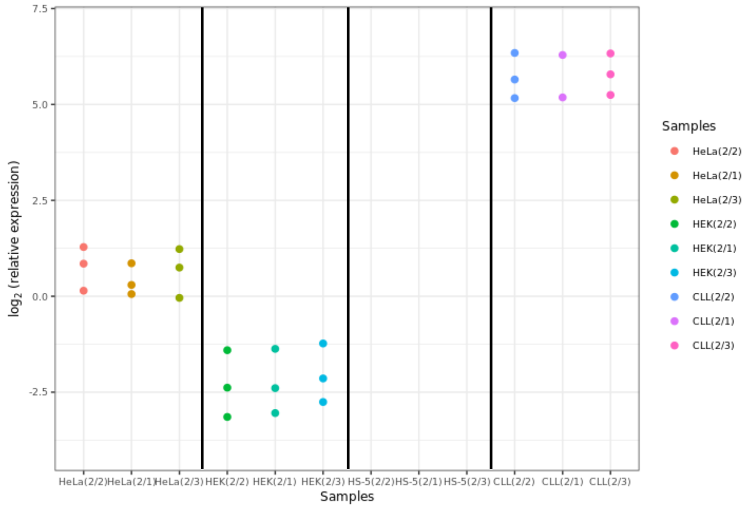

(c)

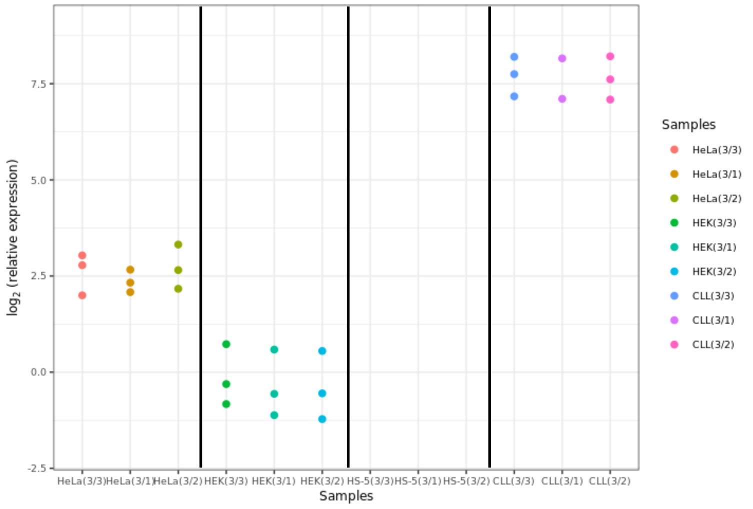

Figure S19: Comparison of relative expression of *CXCR4* in different cell lines and in CLL cells from patients when the reference genes (*HPRT* and *PPIA*) were measured either in the same run as *CXCR4* or in different runs: (a) *CXCR4* measured in run 1; (b) *CXCR4* measured in run 2; (c) *CXCR4* measured in run 3. Each of the panel subsections divided by vertical lines summarizes results for one cell type. The leftmost result in each subsection pertains to the case when *CXCR4* and the RGs were measured on the same plate; the middle and right results pertain to cases when RGs were measured on one of the other two plates. Each particular combination is denoted by numbers in brackets after the sample names, where the numerator is the number of the run in which *CXCR4* was measured and the denominator is the number of the run in which the RGs were measured.

(a)

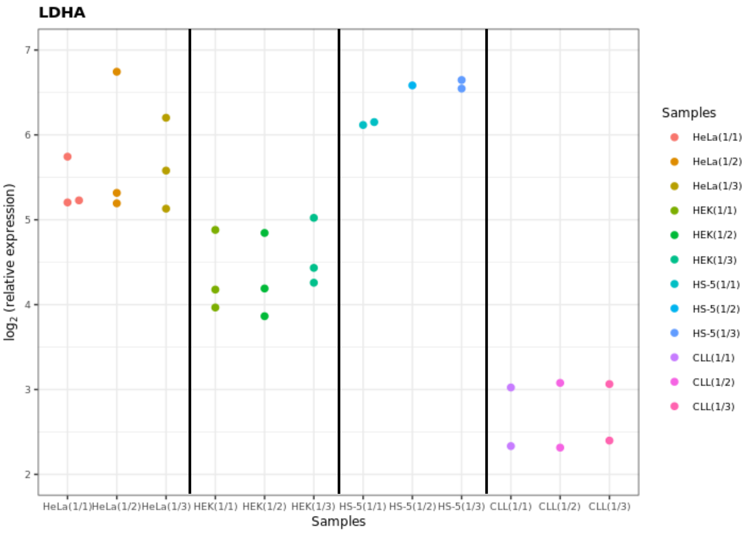

(b)

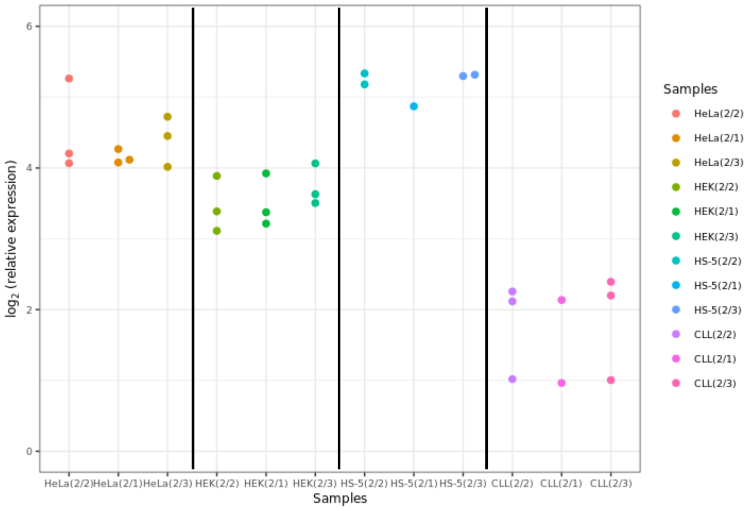

(c)

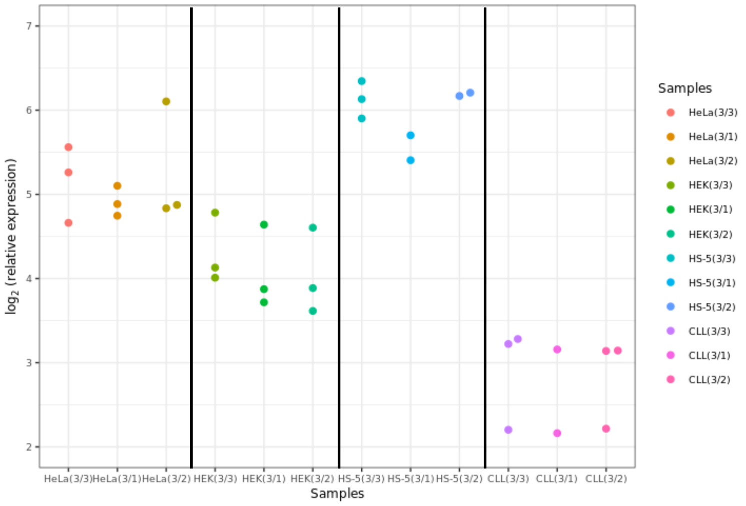

Figure S20: Comparison of relative expression of *LDHA* in different cell lines and in CLL cells from patients when the reference genes (*HPRT* and *PPIA*) were measured either in the same run as *LDHA* or in different runs: (a) *LDHA* measured in run 1; (b) *LDHA* measured in run 2; (c) *LDHA* measured in run 3. Each of the panel subsections divided by vertical lines summarizes results for one cell type. The leftmost result in each subsection pertains to the case when *LDHA* and the RGs were measured on the same plate; the middle and right results pertain to cases when RGs were measured on one of the other two plates. Each particular combination is denoted by numbers in brackets after the sample names, where the numerator is the number of the run in which *LDHA* was measured and the denominator is the number of the run in which the RGs were measured.

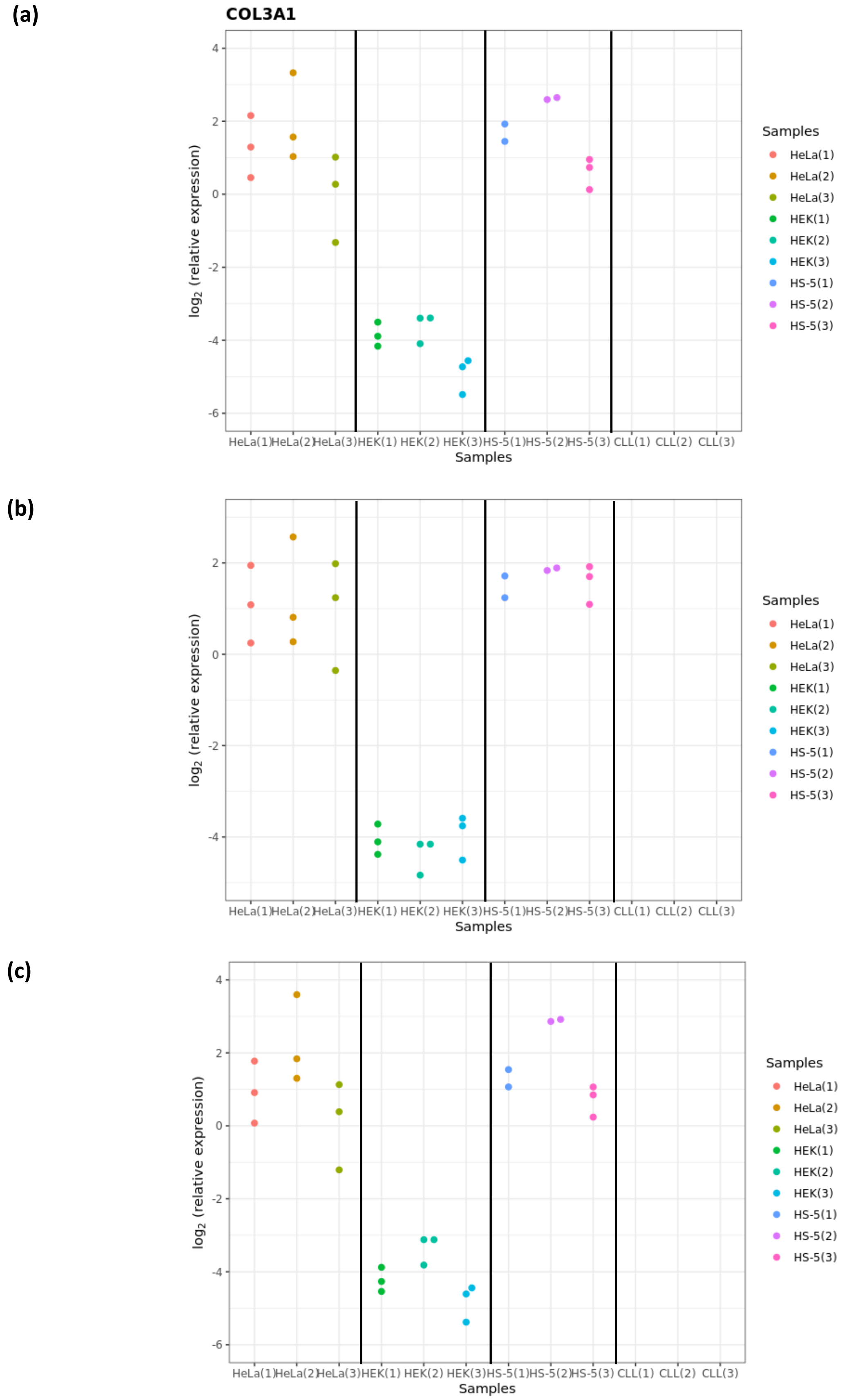

Figure S21: Inter-run comparison of relative expression of *COL3A1* in different cell lines and in CLL cells from patients: (a) without inter-run calibration; (b) with inter-run calibration on a per plate and target basis using Factor qPCR; (c) with inter-run calibration on a per plate basis using Factor qPCR. Results from each particular run are denoted by its number in brackets after sample names. *HPRT* and *PPIA* were used as reference genes.

(a)

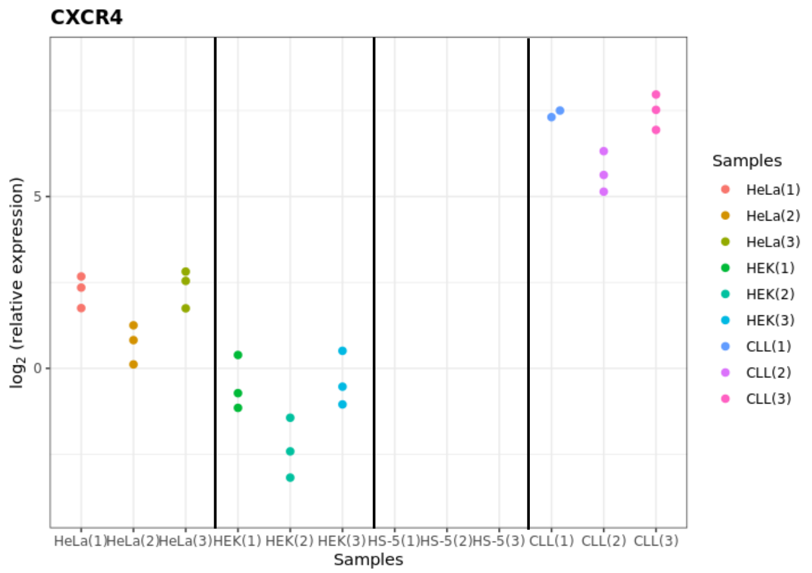

(b)

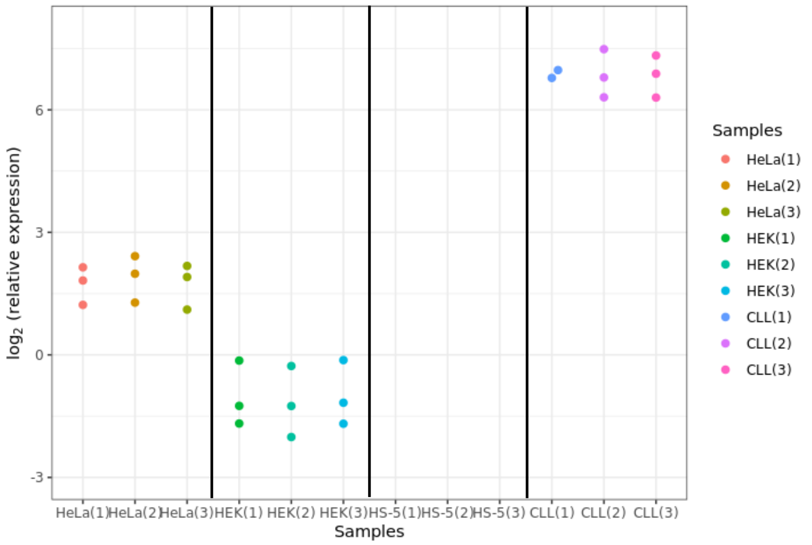

(c)

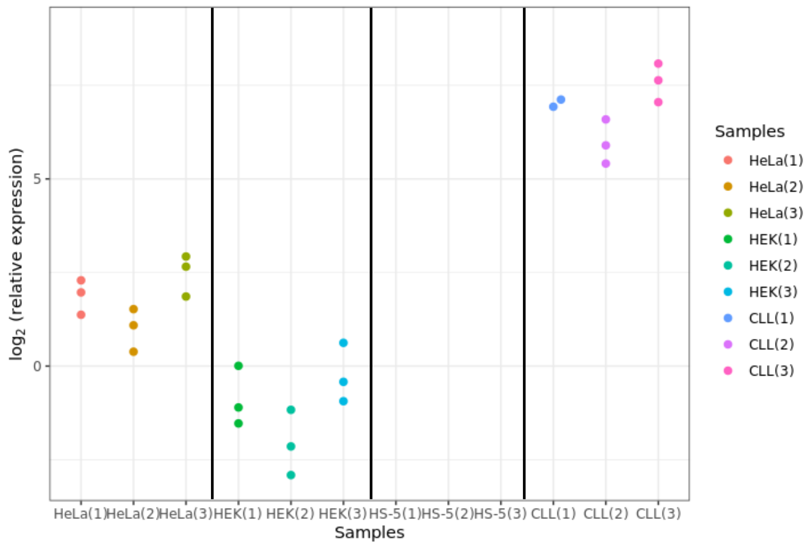

Figure S22: Inter-run comparison of relative expression of *CXCR4* in different cell lines and in CLL cells from patients: (a) without inter-run calibration; (b) with inter-run calibration on a per plate and target basis using Factor qPCR; (c) with inter-run calibration on a per plate basis using Factor qPCR. Results from each particular run are denoted by its number in brackets after sample names. *HPRT* and *PPIA* were used as reference genes.

(a)

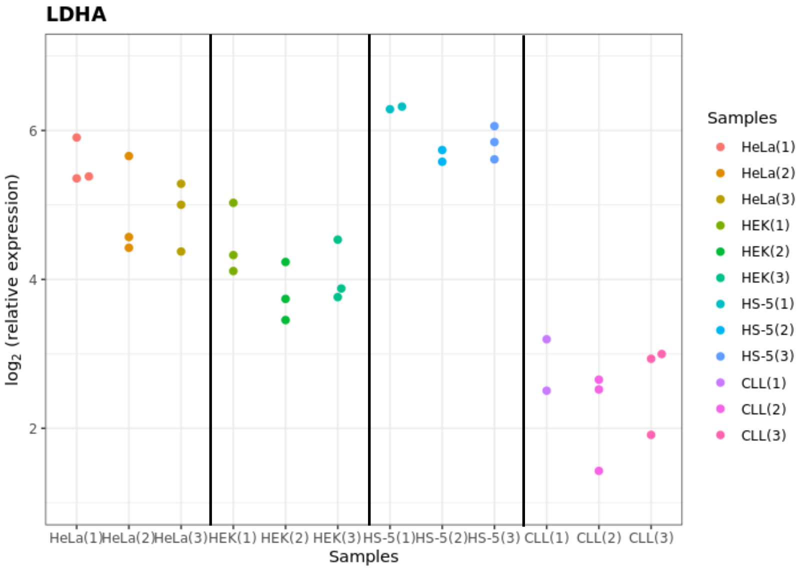

(b)

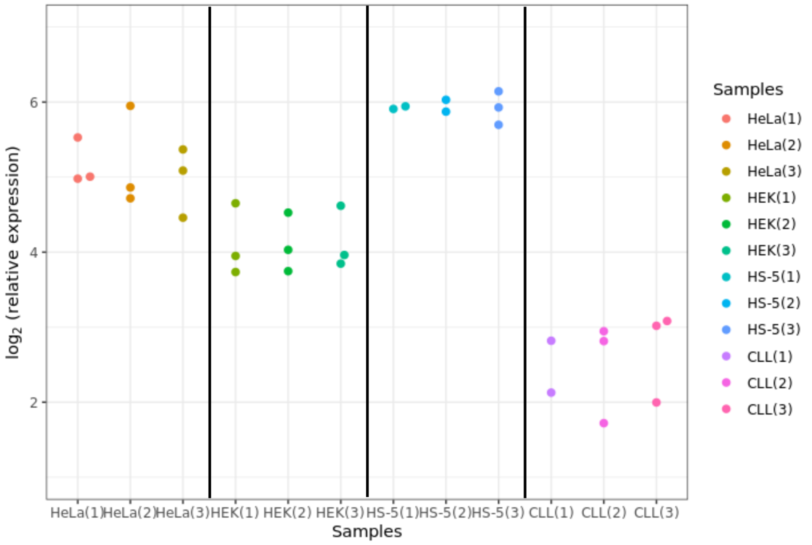

(c)

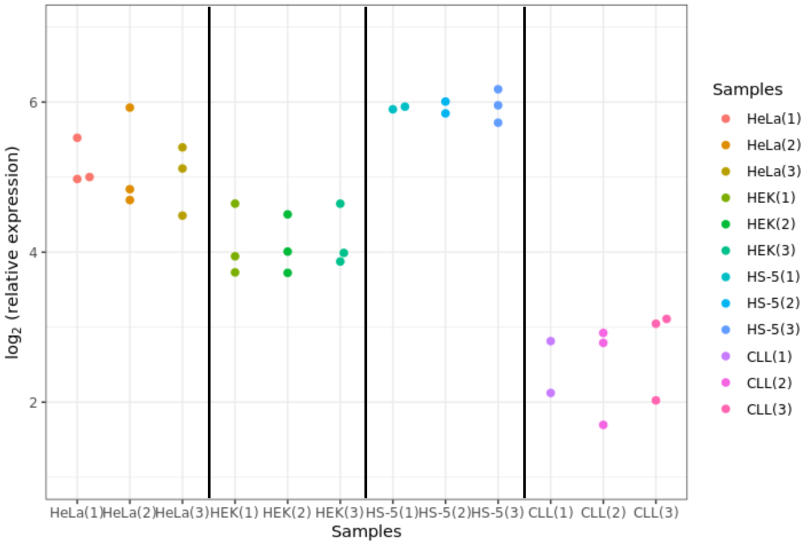

Figure S23: Inter-run comparison of relative expression of *LDHA* in different cell lines and in CLL cells from patients: (a) without inter-run calibration; (b) with inter-run calibration on a per plate and target basis using Factor qPCR; (c) with inter-run calibration on a per plate basis using Factor qPCR. Results from each particular run are denoted by its number in brackets after sample names. *HPRT* and *PPIA* were used as reference genes.

6 References

Boggy GJ, Woolf PJ. A Mechanistic Model of PCR for Accurate Quantification of Quantitative PCR Data. PLoS One. 2010;5:e12355.

Bustin SA, Benes V, Garson JA, Hellemans J, Huggett J, Kubista M, et al. The MIQE Guidelines: Minimum Information for Publication of Quantitative Real-Time PCR Experiments. Clin Chem. 2009;55:611-22.

Hellemans J, Mortier G, De Paepe A, Speleman F, Vandesompele J. qBase relative quantification framework and software for management and automated analysis of real-time quantitative PCR data. Genome Biol. 2007;8:R19.

Hui K, Feng ZP. Efficient experimental design and analysis of real-time PCR assays. Channels (Austin). 2013;7:160-70.

Pfaffl MW. A new mathematical model for relative quantification in real-time RT-PCR. Nucleic Acids Res. 2001;29:e45.

Rieu I, Powers SJ. Real-time quantitative RT-PCR: design, calculations, and statistics. Plant Cell. 2009;21:1031-3.

Ruijter JM, Ramakers C, Hoogaars WM, Karlen Y, Bakker O, van den Hoff MJ., Moorman AF. Amplification efficiency: linking baseline and bias in the analysis of quantitative PCR data. Nucleic Acids Res. 2009;37:e45.

Ruijter JM, Pfaffl MW, Zhao S, Spiess AN, Boggy G, Blom J, et al. Evaluation of qPCR curve analysis methods for reliable biomarker discovery: bias, resolution, precision, and implications. Methods. 2013;59:32-46.

Ruijter JM, Ruiz Villalba A, Hellemans J, Untergasser A, van den Hoff MJ. Removal of between-run variation in a multi-plate qPCR experiment. Biomol Detect Quantif. 2015;30:10-4.

Rutledge RG. A Java Program for LRE-Based Real-Time qPCR that Enables Large-Scale Absolute Quantification. PLoS One. 2011;6:e17636.

Tellinghuisen J, Spiess AN. Comparing real-time quantitative polymerase chain reaction analysis methods for precision, linearity, and accuracy of estimating amplification efficiency. Anal Biochem. 2014;449:76-82.

Vandesompele J, De Preter K, Pattyn F, Poppe B, Van Roy N, De Paepe A, Speleman F. Accurate normalization of real-time quantitative RT-PCR data by geometric averaging of multiple internal control genes. Genome Biol. 2002;3:RESEARCH0034.
